# Transcranial Pulsed Current Stimulation and Social Functioning in Children With Autism: A Randomized Clinical Trial

**DOI:** 10.1001/jamanetworkopen.2025.5776

**Published:** 2025-04-21

**Authors:** Zhenhuan Liu, Sandra Zhong, Roger C. M. Ho, Xuguang Qian, Yan Tang, Hui Tian, Chuntao Zhang, Nuo Li, Yong Zhao, Yuqiong Zhang, Huituan Liu, Meifeng Wu, Yingjie Zhan, Min Li, Zhihai Lv, Fengyi Hao, Wilson Tam, Jeremy Lin Bingyuan, Alvaro Pascual-Leone

**Affiliations:** 1 Department of Paediatrics, Nanhai Maternity and Children’s Hospital Affiliated to Guangzhou University of Chinese Medicine, Foshan, Guangdong, China; 2 Institute of Psychiatry, Psychology & Neuroscience, King’s College London, London, UK; 3 AscenZion Neuromodulation Co Pte Ltd, Singapore; 4 Institute for Health Innovation and Technology (iHealthtech), National University of Singapore, Singapore; 5 Division of Life Sciences (LIFS), Hong Kong University of Science and Technology, Clear Water Bay, Hong Kong; 6 Yunnan University of Chinese Medicine, Kunming, Yunnan Province, China; 7 Department of Paediatrics, Dongguan Maternal and Child Health Hospital, Dongguan, Guangdong, China; 8 Department of Paediatrics, Guangzhou Angel Children Hospital, Guangzhou, Guangdong, China; 9 Department of Paediatrics, Zhanjiang Maternal and Child Health Hospital, Zhanjiang, Guangdong, China; 10 Department of Paediatrics, Meixian District Hospital of Chinese Medicine, Meizhou, Guangdong, China; 11 Department of Paediatrics, Shenzhen Luogang Maternal and Child Health Hospital, Guangdong, China; 12 Sleep Medicine Translational Neuroscience Center, West China Hospital of Sichuan University, Chengdu, China; 13 Alice Lee Centre for Nursing Studies, National University of Singapore, Singapore; 14 Department of Paediatrics, Yong Loo Lin School of Medicine, National University of Singapore, Singapore; 15 Khoo Teck Puat-National University Children’s Medical Institute, National University Health System, Singapore; 16 Hinda and Arthur Marcus Institute for Aging Research, Deanna and Sidney Wolk Center for Memory Health, Hebrew SeniorLife, Boston, Massachusetts; 17 Department of Neurology, Harvard Medical School, Boston, Massachusetts

## Abstract

**Importance:**

Transcranial pulsed current stimulation (tPCS) may improve social functioning and sleep disorders in children with autism spectrum disorder (ASD). Prior trials have been limited by small sample sizes, single-center designs, and often a lack of sham controls.

**Objective:**

To examine the safety and efficacy of tPCS in improving social functioning and sleep disorders in children with ASD.

**Design, Setting, and Participants:**

This multicenter, double-blind, 2-armed, sham-controlled randomized clinical trial, conducted from May 1, 2022, through November 30, 2023, assessed children aged 3 to 14 years with ASD at 8 medical centers in China.

**Interventions:**

Participants underwent daily 20-minute sessions of active tPCS (0.7 mA) or sham tPCS (brief 0.7 mA ramp-up and ramp-down) for 20 sessions over 4 weeks with anode over the right cerebellar hemisphere and cathode over the left dorsolateral prefrontal cortex (12.56-cm^2^-circular, 4-cm-diameter circular electrodes). Each day after tPCS, all participants received 1 hour of standard therapy.

**Main Outcomes and Measures:**

Social functioning was assessed using the Autism Treatment Evaluation Checklist as the primary outcome. Secondary outcomes included the Autism Behavior Checklist and the Childhood Sleep Habits Questionnaire.

**Results:**

A total of 312 participants (155 in the active group and 157 in the sham group; 248 [79.5%] boys; mean [SD] age, 5.1 [1.6] years; 276 [88.5%] aged 3-6 years and 36 [11.5%] aged 7-14 years) completed the trial. After 20 sessions, the mean Autism Treatment Evaluation Checklist total score improved by 4.13 points (5.8%) in the sham tPCS group and 7.17 points (10.7%) in the active tPCS group. Analysis of covariance showed significantly greater improvement in the active tPCS group (difference, −3.50; 95% CI, −5.56 to −1.43; *P* < .001). Both treatments were well tolerated.

**Conclusions and Relevance:**

In this randomized clinical trial of prefrontal-cerebellar tPCS in children aged 3 to 14 years with ASD, 20 sessions over 4 weeks improved social functioning and sleep. These findings suggest that tPCS may serve as a viable nonpharmacologic alternative for ASD.

**Trial Registration:**

Chinese Clinical Trial Registry Identifier: ChiCTR2200059118

## Key Points

**Question:**

Is prefrontal-cerebellar transcranial pulsed current stimulation (tPCS) a safe and efficacious therapy for social functioning in children with autism spectrum disorder (ASD)?

**Findings:**

In this multicenter, double-blind, sham-controlled randomized clinical trial, 312 children aged 3 to 14 years with ASD underwent 20 sessions of prefrontal-cerebellar tPCS over 4 weeks. Treatment results demonstrated significantly improved social functioning compared with sham stimulation and no association of tPCS with serious adverse events.

**Meaning:**

These findings suggest that prefrontal-cerebellar tPCS is a safe and efficacious treatment to improve social functioning in children aged 3 to 14 years with ASD.

## Teaser

This randomized clinical trial of children with autism spectrum disorder examines whether transcranial pulsed current stimulation improves social functioning and sleep disorders.

## Introduction

Autism spectrum disorder (ASD) is a common, heterogenous neurodevelopmental disorder.^1^ According to a 2020 survey in the US,^2^ there is 1 case of ASD in every 36 children aged 8 years. Clinical presentation is often characterized by deficits in social interaction, communication, and stereotyped behaviors.^1^ Additionally, sleep dysfunctions are frequent in ASD^3,4,5^ and can exacerbate daytime behavioral challenges,^6^ leading to caretaker burnout.

Animal and human studies have established genetic risk factors^7^ and environmental influences^8^ as significant contributors to the development of ASD; however, the precise causes remain unclear. The consensus is that ASD symptoms are caused by impaired brain function associated with aberrant functional connectivity,^9,10^ abnormal plasticity,^11^ and disruptions in excitation and inhibition (E/I) balance in cortical circuits^12,13^ that characterize dysfunctional neurocognitive reorganization during development, negatively impacting social cognition,^14^ language comprehension,^15^ and executive function.^16^ Currently, there is no known cure for ASD. Standard of care mainly encompasses a combination of behavioral therapies^17^ and antipsychotic medications to manage challenging behaviors.^18^ However, pharmacologic interventions may lead to adverse effects,^19^ while behavioral therapies are expensive and time-consuming, which affects treatment adherence.^20^ Meta-analyses of these interventions show controversial effectiveness^21,22^; thus, there is a pressing need for interventions that can augment their efficacy or the development of novel treatments.

A promising approach is transcranial pulsed current stimulation (tPCS),^23,24,25,26,27,28,29,30^ a type of noninvasive brain stimulation technique that modulates cortical activity via surface scalp electrodes by delivering low-intensity pulsed currents (<2 mA) at a predetermined frequency to the cortex. tPCS modifies electroencephalography band power in a frequency-specific manner,^30^ modulates functional connectivity,^27^ facilitates interhemispheric coherence,^25^ and enhances cortical plasticity.^28^ In Singapore, 400-Hz tPCS^24^ has received regulatory approval for treatment of spasticity in children with cerebral palsy, supporting its safety in pediatric populations and encouraging the exploration in other conditions. The effects of tPCS in ASD have not been investigated, but studies involving transcranial direct current stimulation (tDCS)^31^ targeting the prefrontal cortex and cerebellum have shown positive effects on core ASD symptoms.^32,33,34,35,36^ Compared with tDCS, tPCS incorporates higher stimulation frequencies that encounter less scalp impedance^37^ and can induce neurophysiologic responses at lower intensities via temporal summation.^24^

We hypothesized that prefrontal-cerebellar tPCS, combined with standard therapy, would enhance social functioning in children aged 3 to 14 years with ASD. As a secondary outcome measure, we proposed that tPCS would improve sleep dysfunctions in ASD, supported by evidence linking the prefrontal and cerebellar regions to sleep regulation^38,39^ and previous studies^32,40^ showing improved sleep outcomes using similar montages.

## Methods

### Trial Design

We conducted a multicenter, double-blind, sham-controlled randomized clinical trial of an evaluable population involving 8 hospitals in China (Guangzhou University of Traditional Chinese Medicine affiliated Nanhai Maternity and Children’s Hospital, Dongguan City Maternal and Child Health Hospital, Zhanjiang City Maternal and Child Health Hospital, Luoding City Maternal and Child Health Hospital, Meizhou City Meixian District Traditional Chinese Medicine Hospital, Guangzhou Angel Children’s Hospital, Shenzhen Luogang District Maternal and Child Health Hospital, and Foshan City Sichuang Special Needs Education School and Hospital). Centralized ethics approval was obtained from the Nanhai Maternity and Children’s Hospital Research Ethics Committee. The study was performed in compliance with ethical standards established in accordance with the Declaration of Helsinki.^41^ Written informed consent was obtained from parents or legal guardians of all participants. Additionally, children 6 years or older who were capable of understanding the nature of the study provided their assent. The study adhered to the Standard Protocol Items: Recommendation for Interventional Trials (SPIRIT)^42^ and the Consolidated Standards of Reporting Trials (CONSORT) reporting guidelines. The trial protocol can be found in Supplement 1.

### Participants

Eligible participants were children aged 3 to 14 years diagnosed with ASD according to *Diagnostic and Statistical Manual of Mental Disorders* (Fifth Edition) criteria^1^ and an IQ of 35 higher on the Wechsler Preschool and Primary Scale of Intelligence, Fourth Edition^43^ for those aged 3 to 6 years and Wechsler Intelligence Scale for Children, Fourth Edition^44^ for those aged 7 to 14 years. The recruitment period was from May 1, 2022, through November 30, 2023. Nonverbal subtests were used for nonverbal participants to ensure accurate evaluation of intellectual potential. Diagnosis of ASD was established by child psychiatrists, and IQ assessment by educational specialists. Exclusion criteria included epilepsy; craniotomy; implanted devices; severe psychiatric conditions (eg, psychosis, schizophrenia); active scalp infection; obstructive sleep apnea; active benzodiazepine, risperidone, or haloperidol use; or prior noninvasive brain stimulation. Centralized randomization across the 8 centers (1:1 to the active tPCS or sham tPCS group) was performed using SPSS software, version 27.0 (IBM Inc). Participants received sealed envelopes in order of their visits, which on opening determined their group allocation. The randomization list was kept confidential by an uninvolved administrator.

### Intervention

#### tPCS

All participants underwent 20 daily sessions of active tPCS or sham tPCS on consecutive weekdays over 4 weeks, performed by therapists blinded to group allocation. We used a transcranial pulsed current stimulator (model YQ-D1111, YIQI Biotechnology Ltd) for 400-Hz tPCS. Before treatment, a pair of 12.56-cm^2^-circular silver-fiber cotton electrodes were presoaked in 0.9% sodium chloride solution and positioned on the participants’ scalps using a fitted head strap. The cathode electrode was placed over the left dorsolateral prefrontal cortex at the F3 position (electroencephalography 10-20 international system) located using the Beam-F3 method^45^ to suppress neural hyperexcitability.^46^ The anode electrode was positioned over the right cerebellar hemisphere, 1 cm below and 4 cm to the right of the inion to enhance frontal-posterior functional connectivity, identified in research as deficient in individuals with ASD^47^ and correlated with symptom severity.^47^ Each stimulation session lasted 20 minutes. In the active tPCS group, there was a 10-second ramp-up to 0.7 mA, maintained throughout, and a 10-second ramp-down at the end. The sham tPCS group experienced a 10-second ramp-up to 0.7 mA followed by a 10-second ramp-down to 0 mA for the remainder of the session. We used tESPlan, version 4.0 (Neurophet Inc) to construct an individualized 3-dimensional head model of a 6-year-old child based on T1-weighted magnetic resonance imaging to estimate the tPCS-induced electric field distribution, which was approximately 0.15 V/m beneath the cathode (F3) and 0.20 V/m beneath the anode (right cerebellar) (eFigure 1 in Supplement 3).

#### Concomitant Therapy

Immediately after tPCS, participants in both groups were given a 1-hour behavioral therapy administered by blinded occupational therapists. This therapy included applied behavior analysis therapy,^48^ structured education,^49^ play-based therapy,^50^ and speech therapy,^51^ rotated for each participant such that each therapy was administered 5 times during the 20-session treatment course. These therapies constitute routine clinical care for ASD in China,^52^ with modest efficacy evidenced in prior studies.^21,53^ The therapies were included to provide a consistent foundation for all study participants.

#### Safety Monitoring

The safety of both the active tPCS and sham tPCS interventions were monitored throughout the 20 sessions by experienced clinical staff in all centers. Urine testing was performed before and after the 20 sessions to assess for unintended metabolic changes. Participants were instructed to report other adverse events experienced during or after treatment that were not included in the list of serious adverse events.

### Outcome Measures

The primary outcome measure was the Autism Treatment Evaluation Checklist (ATEC),^54^ a questionnaire that includes 77 items divided into 4 subscales, including speech, language, and communication; sociability; sensory and cognitive awareness; and health, physical condition, and behavior, yielding a maximum total score of 179, with higher scores indicating more severe symptoms. The secondary outcome measures were the Autism Behavior Checklist (ABC)^55^ and the Childhood Sleep Habits Questionnaire (CSHQ).^56^ The ABC^55^ is a screening tool with 57 items across 5 subscales: sensory, social relating, body and object use, language, and social self-help, with higher scores indicating more severe symptoms. A total score less than 53 is negative, 53 to 67 is positive, and 68 or higher supports an autism diagnosis. The CSHQ^56^ is a parent-report tool to assess children’s sleep patterns through 50 items across 8 sleep dimensions. Each item is rated on a 3-point Likert scale based on frequency, and a score of 41 or higher suggests probable sleep problems. All 3 assessments were administered by 2 evaluators each, who were qualified pediatricians or occupational therapists, at baseline and after 20 sessions of tPCS. Participants’ final scores were the mean score from both raters.

### Masking

The active and sham tPCS devices were visually and operationally indistinguishable. They were located in separate treatment rooms with identical setup at each site to prevent group interaction. Participants and their parents returned to the same room for all 20 sessions. All participants were tPCS naive and informed that any sensations experienced were typical. Room assignments for sham or active treatments were randomized. Outcome assessors were masked to group allocation because they were not involved in randomization, allocation, or treatment. Study staff administering tPCS treatments were unaware of group allocation and treated 1 group exclusively. Despite the absence of a formal blinding evaluation, these measures minimized the risk of deducing group allocation because the chance of comparing treatment responses and adverse effects between groups was remote.

### Statistical Analysis

All statistical analyses were performed using SPSS Statistics for Windows, version 27.0 (IBM Inc) and included all participants in a completer analysis. Data normality was assessed with the Shapiro-Wilk test. At baseline, *t* tests and χ^2^ tests were used to examine whether there was any difference in the continuous and categorical variables between the 2 groups. Analyses of covariance (ANCOVAs) were used to examine the difference between the intervention and control groups for both primary and secondary outcomes after the intervention, after accounting for the baseline values. The Bonferroni correction was applied to control for multiple comparisons. A 2-sided *P* < .05 was considered statistically significant.

## Results

### Participants

A total of 476 potential participants from inpatient or community outreach were assessed for eligibility (Figure). Of these, 340 met the criteria and were randomized 1:1 into the 2 groups (170 per group). Before treatment began, 14 participants were excluded due to the use of antipsychotics or nonapproved concomitant treatments. After treatment commenced, 11 participants discontinued due to travel restrictions, and 3 participants left without specifying a reason.

**Figure.**
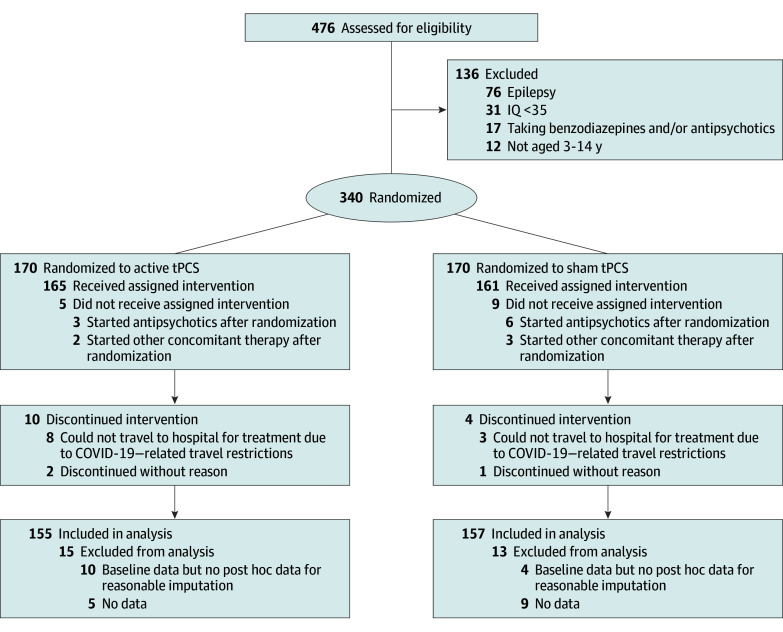
Flow Diagram tPCS indicates transcranial pulsed current stimulation.

In total, 312 participants (155 in the active tPCS group and 157 in the sham group) completed the 20-session treatment and were analyzed (248 [79.5%] boys and 64 [20.5%] girls; mean [SD] age, 5.1 [1.6] years; 276 [88.5%] aged 3-6 years and 36 [11.5%] aged 7-14 years). Results of baseline comparison of the demographics and clinical characteristics of the participants between groups are given in Table 1. There were no notable differences in the variables, indicating similar severity in social functioning and sleep disorders between groups.

**Table 1.** Participants’ Demographic and Clinical Characteristics at Baseline

Characteristic	Sham group (n = 157)	Active group (n = 155)
Demographic characteristics		
Age, mean (SD), y	5.1 (1.5)	5.1 (1.7)
Age group, No. (%)		
Preschool (3-6 y)	140 (89.2)	136 (87.7)
School age (7-14 y)	17 (10.8)	19 (12.3)
Sex, No. (%)		
Male	121 (77.1)	127 (81.9)
Female	36 (22.9)	28 (18.1)
Clinical characteristics, mean (SD)		
Speech, language, and communication score	17.20 (6.30)	17.30 (6.15)
Sociability score	18.65 (6.93)	17.61 (6.40)
Sensory and cognitive awareness score	18.45 (6.57)	17.06 (6.22)
Physical, health, and behavior score	16.84 (8.19)	15.30 (7.79)
ATEC scores		
Total	71.13 (22.65)	67.27 (20.79)
Sensory	10.83 (5.64)	12.28 (5.69)
Relating	17.82 (6.38)	17.77 (4.66)
Body and object use	11.24 (6.43)	11.54 (6.36)
Language	16.31 (5.79)	16.88 (5.17)
Social self-help	11.70 (4.56)	11.70 (4.34)
ABC scores		
Total	67.90 (18.22)	70.20 (16.6)
Bedtime resistance	10.66 (2.94)	9.99 (2.78)
Sleep anxiety	4.62 (2.81)	5.16 (2.48)
Sleep duration	5.53 (1.91)	5.47 (2.03)
Parasomnia	11.67 (4.77)	11.37 (4.74)
Night wakening	5.36 (2.31)	5.39 (2.33)
Sleep disordered breathing	4.87 (2.44)	4.64 (2.25)
Daytime sleepiness	13.57 (4.80)	13.39 (4.34)
Sleep onset delay	1.9 2 (0.73)	1.95 (0.82)
CSHQ total score	58.20 (16.20)	57.35 (15.07)

Abbreviations: ABC, Autism Behavior Checklist; ATEC, Autism Treatment Evaluation Checklist; CSHQ, Childhood Sleep Habits Questionnaire.

### Outcomes and Estimation

After treatment, the mean (SD) ATEC total score was 67.0 (22.3) in the sham tPCS group and 60.1 (20.0) in the active tPCS group, with mean (SD) score reductions of −4.13 (8.7) points (5.8%) and −7.17 (10.5) points (10.7%), respectively. ANCOVA revealed significantly greater improvement in the active tPCS group compared with the sham tPCS group (difference, −3.50; 95% CI, −5.56 to −1.43; *P* < .001; effect size, 0.04), driven by a significant difference in the sociability subdomain (difference, −1.72; 95% CI, −2.57 to −0.88; *P* < .001; effect size, 0.05). Other ATEC subdomains showed no significant differences.

For secondary outcome measures, after treatment, the mean (SD) ABC total score was 66.3 (18.1) for the sham tPCS group and 64.2 (15.7) for the active tPCS group, with mean (SD) score reductions of −1.6 (8.6) points (2.4%) and −6.0 (7.7) points (8.5%), respectively. ANCOVA revealed a significantly greater improvement in the active tPCS group (difference, −4.00; 95% CI, −5.75 to −2.26; *P* < .001; effect size, 0.06), underscored by a significant difference in the sensory subdomain (difference, −0.86; 95% CI, −1.50 to −0.22; *P* = .009; effect size, 0.02). None of the other ABC subdomains were significant. In assessing sleep changes, the posttreatment mean (SD) CSHQ total score was 56.3 (16.7) for the sham tPCS group and 53.2 (17.4) for the active tPCS group, with mean (SD) score reductions of −1.9 (3.9) points (3.3%) in the sham tPCS group and −4.2 (5.2) points (7.3%) in the active tPCS group. ANCOVA revealed greater improvement in the active tPCS group (difference, −2.19; 95% CI, −3.20 to −1.19; *P* < .001; effect size, 0.06), particularly in the daytime sleepiness (difference, −0.85; 95% CI, −1.30 to −0.39; *P* < .001; effect size, 0.04). None of the other CSHQ subdomains were significant (Table 2). Exploratory post hoc linear regression indicated that the improvement in CSHQ total score was not significantly associated with the improvement (reduction) in ATEC total score of the participants in either group (active tPCS group: *R*^2^ linear = 0.02, *P* = .06; sham tPCS group: *R*^2^ linear = 0.005, *P* = .39) (eFigure 2 in Supplement 2).

**Table 2.** Posttreatment Analysis of Covariance for ATEC, ABC, and CSHQ

Outcome variable	Without baseline adjustment, mean (SD)		With baseline adjustment				
	Sham group scores	Active group scores	Sham group scores, mean (SD)	Active group scores, mean (SD)	Adjusted difference, mean (95% CI)	*P* value	Effect size
Speech, language, and communication	16.3 (6.4)	15.7 (6.0)	16.32 (4.93)	15.62 (4.95)	0.70 (−0.08 to 1.48)	.08	0.01
Sociability	17.5 (6.5)	15.0 (5.7)	17.13 (5.35)	15.41 (5.39)	1.72 (0.88 to 2.57)	<.001	0.05
Sensory and cognitive awareness	17.3 (6.5)	15.6 (6.0)	16.74 (5.05)	16.12 (5.07)	0.62 (−0.18 to 1.42)	.13	0.01
Physical, health, and behavior	16.0 (7.6)	14.0 (7.2)	15.33 (5.02)	14.52 (5.05)	0.81 (0.01 to 1.60)	.047	0.01
ATEC score							
Total	67.0 (22.3)	60.1 (20.0)	65.35 (13.0)	61.85 (13.1)	3.50 (1.43 to 5.56)	<.001	0.04
Sensory	10.5 (5.6)	10.9 (5.2)	11.12 (4.04)	10.26 (4.08)	0.86 (0.22 to 1.50)	.009	0.02
Relating	17.4 (6.2)	16.5 (4.4)	17.34 (4.65)	16.47 (4.68)	0.87 (0.13 to 1.60)	.02	0.02
Body and object use	10.8 (6.4)	10.3 (6.1)	10.96 (4.72)	10.14 (4.73)	0.82 (0.07 to 1.56)	.03	0.02
Language	16.3 (5.9)	15.7 (5.1)	16.51 (5.12)	15.47 (5.16)	1.04 (0.23 to 1.85)	.01	0.02
Social self-help	11.2 (4.2)	11.0 (4.2)	11.22 (3.67)	10.94 (3.69)	0.28 (−0.30 to 0.86)	.35	0.00
ABC score							
Total	66.3 (18.1)	64.2 (15.7)	67.23 (11.0)	63.23 (11.1)	4.00 (2.26 to 5.75)	<.001	0.06
Bedtime resistance	10.1 (2.9)	9.4 (3.2)	9.77 (2.54)	9.70 (2.56)	0.07 (−0.33 to 0.48)	.73	0.00
Sleep anxiety	4.5 (2.7)	4.6 (2.1)	4.68 (1.55)	4.36 (1.57)	0.32 (0.08 to 0.57)	.01	0.02
Sleep duration	5.5 (1.8)	5.1 (2.1)	5.44 (1.87)	5.11 (1.89)	0.33 (0.04 to 0.63)	.03	0.02
Parasomnia	11.4 (5.0)	10.6 (5.2)	11.20 (2.23)	10.74 (2.24)	0.46 (0.11 to 0.81)	.01	0.02
Night wakening	5.2 (2.3)	5.0 (2.3)	5.16 (1.31)	5.03 (1.31)	0.13 (−0.08 to 0.34)	.22	0.01
Sleep disordered breathing	4.8 (2.5)	4.5 (2.3)	4.69 (0.72)	4.65 (0.72)	0.04 (−0.07 to 0.16)	.49	0.00
Daytime sleepiness	13.2 (4.7)	12.2 (5.0)	13.12 (2.88)	12.27 (2.90)	0.85 (0.39 to 1.30)	<.001	0.04
Sleep onset delay	1.8 (0.7)	1.8 (0.8)	1.84 (0.72)	1.81 (0.72)	0.03 (−0.08 to 0.15)	.59	0.00
CHSQ total score	56.3 (16.7)	53.2 (17.4)	55.88 (6.38)	53.69 (6.43)	2.19 (1.19 to 3.20)	<.001	0.06

Abbreviations: ABC, Autism Behavior Checklist; ATEC, Autism Treatment Evaluation Checklist; CSHQ, Childhood Sleep Habits Questionnaire.

Exploratory analyses, which used a 10% or greater reduction in posttreatment scores as a benchmark for clinically meaningful change, revealed that 84 of 155 participants (54.2%) in the active tPCS group achieved this outcome with the ATEC total score compared with 48 of 157 (30.6%) in the sham tPCS group. Similarly, 63 of 155 (40.6%) in the active tPCS group achieved this outcome with the ABC scores, nearly double the 31 of 157 (19.7%) in the sham tPCS group (eTables 2 and 3 in Supplement 2).

### Safety and Adverse Events

Throughout the study, both sham tPCS and active tPCS were well tolerated by all participants. No serious adverse events were reported. Adverse effects reported were limited to mild headache (n = 19) and temporary scalp redness (n = 27) underneath the electrode sites, which self-resolved within 30 minutes after stimulation cessation (eTable 1 in Supplement 2). No participants from either group withdrew from the study due to adverse reactions.

## Discussion

These findings represent the first report, to our knowledge, of the effects of tPCS in children with ASD in an adequately powered, multicenter, double-blind, sham-controlled randomized clinical trial. A total of 312 participants completed the study after being randomly assigned to receive 20 sessions of either sham tPCS or active tPCS (0.7 mA, 20 minutes per session) over 4 weeks in addition to standard therapy. The primary objective of this study was to evaluate the effect of tPCS on social functioning in children with ASD, using the ATEC as the primary outcome measure and the ABC as an alternative assessment. The secondary objective of the study was to investigate the effect of tPCS on sleep dysfunctions in children with ASD, using the CSHQ as the outcome measure.

Results demonstrated that tPCS significantly enhanced social functioning in children with ASD, evidenced by a significant posttreatment difference in baseline-adjusted ATEC total mean scores, which was driven by greater sociability improvements in the active tPCS group. Other ATEC subdomains showed no significant group differences. The ABC assessment confirmed the primary outcome, with posttreatment baseline-adjusted ABC total mean scores showing significant differences between groups. In the ABC subdomain analyses, the active tPCS group had significant improvements in sensory scores and clinically meaningful gains in language abilities compared with the sham tPCS group. However, the language subdomain lost significance after applying a conservative Bonferroni correction, chosen due to the study’s pioneering nature in tPCS research for ASD. It would have remained significant with less stringent corrections. Additionally, the ATEC and ABC were primarily designed to evaluate individual changes over time rather than group comparisons; thus, the small effect sizes observed between groups may still hold clinical significance.

An elevated E/I ratio^13,14^ has been frequently reported in the prefrontal region of individuals with ASD. The observed improvement in social functioning may have been due to the reduction in excitability in the left dorsolateral prefrontal cortex by cathodal tPCS, mediated by both the negative polarity and the effects of the higher-frequency pulses in the tPCS waveform linked to mechanisms such as activation of inhibitory interneurons^57^ and desynchronization of excitatory neuronal activity.^58^ Our findings were consistent with the results of 2 previous studies^33,34^ that used cathodal stimulation (20-minute, 1.5-mA tDCS, 10 sessions) over the left dorsolateral prefrontal cortex in adolescents with ASD and also reported improved social functioning. In one of these studies,^33^ a significant reduction in the θ-band E/I ratio in cortical midline structures related to sociocognitive information processing was demonstrated after treatment.

Cerebellar abnormalities^59,60,61^ and reduced frontal-posterior connectivity^62^ were consistently associated with ASD. A previous study^63^ demonstrated that stimulating Purkinje cells in the cerebellar right crus I enhanced its connectivity with the medial prefrontal connectivity and rescued social impairments in ASD-variant mice. Although the exact mechanism was unclear, we postulated that the anodal 400-Hz tPCS over the right cerebellum contributed to the results by modulating dysfunctional ASD-relevant cerebellar circuits^64^ involved in social^65,66^ and communicative functions,^67^ enhancing contralateral frontal-posterior connectivity^68^ and thereby improving social functioning.

In our study, we assessed the effect of tPCS on sleep in children with ASD as a secondary objective because poor sleep has been associated with worsening ASD symptoms.^3,6^ After treatment, the baseline-adjusted CSHQ total mean score differed significantly between groups, with the active tPCS group showing notable improvement in daytime sleepiness compared with the sham tPCS group. We speculated that the tPCS associated improvement may have been due to the anodal tPCS over the right cerebellum, which could have modulated cerebellar circuits involved in regulating circadian rhythms^69^ and sleep processes.^39,69^ Notably, in other studies involving children with ASD^32^ and patients with bipolar disease,^40^ sleep improvements were also observed when a cerebellar montage was used. Although tPCS was observed to reduce ATEC and CSHQ total scores, in our exploratory post hoc analysis, the improvement in sleep was not significantly associated with the improvement in social functioning, suggesting independent mechanisms. This finding was aligned with a systematic review^70^ that found insufficient evidence to establish a causal relationship between sleep quality and social functioning in children with ASD.

### Limitations

This study has some limitations. A total of 88.5% of our sample consisted of children younger than 7 years (276 of 312 children) who did not consume benzodiazepines, risperidone, or haloperidol. This might limit the generalizability of the results to older children with ASD and those who rely on these medications. Although blinding measures were in place, the study did not include a formal blinding evaluation. The study also lacked resources to perform tests on changes in frontal E/I ratios or functional connectivity that may be linked to behavioral changes. Future studies should investigate the mechanisms behind these effects. Long-term follow-up of participants was not conducted due to logistical challenges with COVID-19 restrictions. Despite these limitations, the study was adequately powered and resulted in significant improvements in social functioning and sleep with a lower dose of 0.7 mA compared with doses of 1 to 1.5 mA used in similar studies,^32,33,34^ potentially enhancing safety for pediatric use.

## Conclusions

In this randomized clinical trial of prefrontal-cerebellar tPCS in children aged 3 to 14 years with ASD, a 20-session, 1-month intervention was demonstrated to be safe and resulted in greater improvement of social functioning and sleep compared with sham stimulation. More than twice as many participants in the active tPCS group than in the sham group achieved meaningful clinical effects. Future phase 4 trials exploring effectiveness in the community setting are warranted.

## Notes

Supplement 1.
Trial Protocol

Supplement 2. **eTable 1.** Summary List of Adverse Events Across the Right Participating Sites **eTable 2.** Summary of Participants Achieving Clinically Meaningful Change in Post-hoc ATEC Total Scores **eTable 3.** Summary of Participants Achieving Clinically Meaningful Change in Post-hoc ABC Total Scores **eFigure 1.** 3D Head Model from T1-Weighted MRI of a 6-Year-Old to Simulate tPCS-Induced Electric Fields **eFigure 2.** Scatterplot Showing CSHQ Total Score Change Predicting ATEC Total Score Change for All Participants Post-Treatment

Supplement 3.
Data Sharing Statement
