# Supplementary material for: Transcranial Pulsed Current Stimulation and Social Functioning in Children With Autism: A Randomized Clinical Trial
Source: JAMA Netw Open. 2025 Apr 21;8(4):e255776. doi: 10.1001/jamanetworkopen.2025.5776 (PMC12013354; doi:10.1001/jamanetworkopen.2025.5776)
Supplement: Supplement 1. — Trial Protocol [file jamanetwopen-e255776-s001.pdf]

Nanhai Maternity and Children's Hospital

Affiliated to Guangzhou University of Chinese Medicine

Research Study Protocol

**PROTOCOL TITLE:**	
Transcranial Pulsed Current Stimulation on Social Functioning in Children with Autism Spectrum Disorder: A multicenter, double-blinded, sham-controlled, randomized clinical trial	
**PROTOCOL VERSION:**	Version 1.1
**PROTOCOL DATE:**	28 Jan 2023
**PRINCIPAL INVESTIGATOR:**	
Dr Zhenhuan Liu<br><br>Senior Consultant Pediatrician, Head of Department, Division of Pediatric Neurorehabilitation, Department of Pediatrics, Nanhai Maternity and Children's Hospital Affiliated to Guangzhou University of Chinese Medicine	
**SITE PRINCIPAL INVESTIGATOR:** Dr Zhenhuan Liu	
**STUDY SITE:**	
Nanhai Maternity and Children's Hospital Affiliated to Guangzhou University of Chinese Medicine	
Dongguan Maternal and Child Health Hospital	
Zhanjiang Maternal and Child Health Hospital	
Luoding Maternal and Child Health Hospital	
Meixian District Hospital of Traditional Chinese Medicine	
Guangzhou Angel Children's Hospital	
Shenzhen Luogang Maternal and Child Health Hospital	
Foshan Sichuang Special Education School and Hospital	
**Co-Investigators:**	**Collaborators**
Dr Tang Yan<br><br>Dr Hui Tian<br><br>Dr Chuntao Zhang<br><br>Dr Nuo Li<br><br>Dr Xuguang Qian<br><br>Dr Yong Zhao<br><br>Dr Huituan Liu<br><br>Dr Yuqiong Zhang<br><br>Dr Meifeng Wu<br><br>Dr Yingjie Zhan<br><br>Dr Min Li<br><br>Dr Zhihai Lv	Prof. Roger C.M. Ho<br><br>Prof. Alvaro Pascual-Leone<br><br>Dr Jeremy Lin Bingyuan<br><br>Dr Wilson Tam<br><br>Dr Fengyi Hao<br><br>Ms. Sandra Zhong

Protocol Deviations

There were two protocol deviations. The Study Protocol deviation was directly associated with the coronavirus disease 2019 (COVID-19) pandemic. During the early part of the trial in 2022, China held on to extremely stringent post-COVID travelling restrictions, as many citizens were still not vaccinated and the country held on to a “zero-infection” policy. This had a tremendous negative impact on the recruitment process of this trial.

Given the circumstances, the Principal Investigator and the study team members estimated the potential drop-out rate may increase to 30% - 40%. After discussing with IRB, we decided to 1) increase the sample size by 40%, from 240 subjects to 340 subjects to account for potential dropouts, and 2) expanded from seven to eight recruiting hospital sites with the addition of Shenzhen Luogang Maternal and Child Health Hospital, in order to facilitate the recruitment of an enlarged sample size. Both protocol deviations were included in this Trial Protocol Version 1.1.

TABLE OF CONTENTS

1. BACKGROUND AND RATIONALE........ 4
1.1. General Introduction........ 4
1.2. Rationale and Justification for the study........4-5
1.3. Rationale for the study purpose........6
1.4. Rationale for the study population........6
1.5. Rationale for the study design 6
2. HYPOTHESIS AND OBJECTIVES........7
2.1. Hypothesis........7
2.2. Primary objective and outcome measures........7
2.3. Secondary objective and outcome measures........7
2.4. Potential risks and benefit........7
3. STUDY POPULATION........ 7
3.1. Number and nature of subjects to be enrolled........ 7
3.2. Study settings........ 8
3.3. Screening visits procedures and recruitment 8
3.4. Diagnostic Criteria........8
3.5. Inclusion criteria........8
3.6. Exclusion criteria........9
3.7. Drop-out criteria........9
3.8. Provision for drop-out cases and handling of missing data........9
4. STUDY DESIGN........ 10
4.1. Randomization: Allocation sequence generation 10
4.2. Randomization: Allocation implementation 11
4.3. Study visits and procedures........11-12
4.4. Discontinuation........13
5. TRIAL MATERIAL........ 13
5.1. Trial product 13
5.2. Trial device characteristics........14
5.3. Electrode materials 14
6. TREATMENT 14
6.1. Rationale for selection of dose 14
6.2. Treatment procedure 14-15
6.3. Specific treatment adherence requirements/ strategies........ 16
6.4. Blinding........16
7. SAFETY MEASUREMENTS........ 16
7.1. Definitions 16-17
7.2. Collection, recording and reporting of serious adverse events to ethics board........ 17
7.3. Safety monitoring plan........17
7.4. Complaint handling........ 17
8. DATA ANALYSIS 17
8.1. Data quality assurance........ 17
8.2. Data entry and storage 17
9. SAMPLE SIZE AND STATISTICAL METHODS 18
9.1. Data quality assurance........18
9.2. Statistical and analytical plans........18
10. DIRECT ACCESS TO SOURCE DATA/ DOCUMENTS........ 19
11.ETHICAL CONSIDERATIONS 19
11.1 Confidentiality of data and patient records........19
12. PUBLICATIONS........19
13. RETENTION OF TRIAL DOCUMENTS 19
14. FUNDING........19
15. REFERENCES........20-25
16. APPENDIX 1: DATA FORM (ATEC)........26
17. APPENDIX 2: DATA FORM (ABC)........27-29
18. APPENDIX 3: DATA FORM (CSHQ)........30-32
19. APPENDIX 4: CONSENT FORM / ASSENT FORM........33-40
20. APPENDIX 5: DATA & SAFETY MONITORING COMMITTEE........41-42

1 Background and Rationale

1.1 General Introduction

Autism spectrum disorders (ASD) is a genetically heterogenous neurodevelopmental disorder characterized by restricted and repetitive behavioural patterns and persistent deficit in social interaction and communication[1]. Sleep dysfunction, in particular chronic insomnia, is a common comorbidity in children with ASD[2,3] which occurs at a significantly higher prevalence of 40-80% as compared to 20-30% in typically developing children[4]. Sleep plays a crucial role in regulating behavior and emotions and deprivation of sleep can exacerbate daytime behavioral challenges in children with ASD[5]. This often leading to caretaker burnout who are sleep deprived themselves and profoundly diminish quality of life for all family members.

In recent years, the incidence rate of ASD reported by various countries has been on the rise, for example, according to the United States' Centers for Disease Control and Prevention survey in 2020, 1 in every 36 children aged 8 years in the United States has ASD[6]. In China at present, there is no nationwide statistical survey on the incidence rate of ASD, however, in the 2017 "Report on the Development of China's Autism Education and Rehabilitation Industry II" , it is conservatively estimated that there are at least 10 million people with ASD among the 1.3 billion population of China, of which 2 million were children with ASD. The situation of rapidly increasing new cases of ASD worldwide is dire.

1.2 Rationale and Justification for the Study

Current guidelines for ASD mainly target patient symptomology and include an individualized treatment solution comprising of a combination of behavioral therapies to improve core symptoms of socio-communicative deficits[7] and pharmacological interventions to manage challenging behaviours and sleep disorders[8]. However, meta-analyses of these interventions show only modest effectiveness with pharmacological interventions invariably causing undesirable side-effects[9,10], in addition, behavioral therapies are time-consuming, costly and often require high involvement of caretakers which reduce adherence over the long term[11]. As there is no intervention shown to cure ASD, a significant portion of individuals with ASD would require lifelong support, which imposes substantial financial and emotional burdens on their families and substantially heightens societal costs. Hence, there is an urgent need for the development of novel treatments that is safe and with greater efficacy in addressing the core and associate symptoms of ASD.

1.2.1 Research findings on pathophysiology of ASD

Decades of research in ASD have revealed that genetic risk factors [12–14] and environmental factors[15] are significant contributors to the susceptibility of a child developing ASD, however the exact causes of ASD remain unclear. The consensus among most researchers is that ASD symptoms are caused by a lack of interhemispheric coherence[16] and impaired cortical functioning , in particular, dysfunctions in functional connectivity of certain brain regions associated with ASD such as the left dorsal lateral prefrontal cortex (L-dLPFC) that controls executive functioning and social cognition[17,18] and the cerebellar that is involved in sociolinguistic processing [19,20] and adaptive predictions in social cognition [21,22]. These brain alterations occur at the cellular and systems level [23] and may lead to disruptions in excitation/inhibition (E/I) balance as supported by various human studies [24–26].

1.2.2 Research on existing knowledge of Transcranial Electrical Stimulation and ASD

In recent years, there has been growing interest in the therapeutic potential of transcranial electrical stimulation (tES) as a novel treatment for several pediatric psychiatric and neurological disorders including ADHD[27,28], cerebral palsy[29,30] , dyslexia[31,32] , and autism spectrum disorder [33–37] because of its ability to induce long-term potentiation (LTP)-like and long-term depression (LTD)-like effects that is thought to mediate neuroplasticity changes [38–40] in the central nervous system.

tES is a form of non-invasive brain stimulation technique that transmits low intensity electrical current (<2mA) to specific areas of the brain via surface electrodes that are placed on the scalp to modulate neuronal excitability and spontaneous firing rates[41].There are different modes of tES according to the electrical output waveform[42], among them, tPCS is the most novel type and has gained increasing attention in experimental settings [30,40,43–56] . Furthermore, in clinical use, tPCS with a fixed frequency of 400Hz, has received health regulatory approval in Singapore for the treatment of limb spasticity in pediatric cerebral palsy since 2022[57], marking a significant advancement for the use of tPCS in pediatric-centric healthcare compared to other forms of tES.

tPCS delivers pulsed currents at a predetermined frequency to the cortex, as opposed to the direct current provided by tDCS. Studies involving tPCS have demonstrated effects on resting state functional connectivity[45,58], reported to facilitate interhemispheric coherence[47,55] and enhance cortical plasticity[39,54]. These neural impact of tPCS may be of relevance to ASD since widespread prefrontal-posterior underconnectivity and atypical information processing between brain regions are well-established in this disorder [59,60]. Furthermore, children with ASD were found to have elevated global excitation-inhibition (E/I) ratio[25] which could serve as a consistent neuromodulatory target in treating ASD across children with heterogenous etiology. EEG bands may act as proxy markers for neural excitation and inhibition[61] and tPCS has been evidenced to increase/decrease EEG band power in a frequency-specific manner[50,55,56,62], indicating its potential therapeutic application in ASD via modulating highly associated brain regions such as the prefrontal cortex and the cerebellar to target E/I imbalances. In fact, a previous study using mouse models of ASD-linked genes demonstrated that modulating activities of cerebellar-prefrontal circuits improved social impairments and inflexible behaviours[63], purported to be due to the activation of GABAergic Purkinje cells in the cerebellar Rcrus1 and the posterior vermis.

Finally, in studies involving children with ASD and transcranial direct current stimulation (tDCS), the most common type of tES used in research, positive improvements in ASD evaluation scores have been reported after stimulation of the L-dLPFC [33,35,64,65], the cerebellar[66] or both the L-dLPFC and the cerebellar [37]. For example, in a study involving 16 ASD children[37], it was reported that 20 sessions of 20 min, 1 mA prefrontal-cerebellar tDCS stimulation resulted in a significant reduction in severity of ASD global symptoms as measured by the Aberrant Behaviour Checklist (ABC) and the Autism Treatment Evaluation Checklist (ATEC) in the active stimulation group compared to the sham stimulation group. In another small open label[67], the quality of sleep and mood in participants was reported to have improved, in addition to a 25% reduction in ABC scores.

1.2.3 Safety of tES in Children

While there are yet no studies done involving tPCS and children with ASD, the safety of tPCS had been

investigated in childhood cerebral palsy[30] and in adult neurological disorders such as Parkinson's disease [68], chronic visceral pain[40] and disorder of consciousness[44], with no adverse events recorded. At present, it is generally considered that an output current between 0-2mA, and the single stimulation time is less than 40 minutes is considered relatively safe [69]. There is no exact guidelines for tES dosing in children although a dose of up to 1mA is typically used. Krishnan C et.al[70] conducted a comprehensive statistical analysis of 48 clinical studies involving more than 513 children and / or adolescents with non-invasive brain stimulation. They found that the most common adverse reaction of tES among people under 18 years of age was skin tingling, with an incidence rate of 11.5%, followed by skin itching, with an incidence rate of 5.8%. These adverse reactions were transient and non-persistent. In terms of cognitive adverse events, treatment-emergent mania and hypomania have been reported in unipolar and bipolar depression treatment in tDCS trials in diseased adults[71–73]. Finally, drug interactions with tDCS have been reported, with antipsychotics with high D2 affinity such as Haloperidol and Risperidone reported to suppress neuromodulation-induced plasticity [74] while benzodiazepines were associated with an increase in depression scores when used in conjunction with tDCS [75].

1.3 Rationale for Study Purpose

Considering the urgent clinical need to improve core and associate symptoms in children with ASD beyond current treatment options, the demonstrated safety profile and efficacy of tPCS in other vulnerable paediatric populations such as cerebral palsy[30] and promising results in improving social functioning in preliminary small-sample studies of tES in children with ASD[33,35–37,67],we aim to investigate the effects of tPCS in addition to standard therapy, in improving social functioning, sleep and challenging behaviours in children with ASD aged 3 to 14 years old, in a higher-powered study.

1.4 Rational for Study Population

ASD symptoms start in childhood and manifest for a lifetime, potential new interventions for ASD to improve social functioning and associated symptoms should start as an early intervention to leverage on enhanced neuroplasticity that occurs during childhood to optimize any treatments effects.

1.5 Rationale for Study Design

The study will be designed as a multi-center, sham-controlled, double-blinded, randomized controlled trial to investigate effects of tPCS in improving social functioning and sleep in children with ASD aged 3-14 years old. Both the intervention and control group will both receive standard of care behavioural therapy for ASD in China (i.e ABA therapy[76], structured teaching[77], play therapy[78] and speech therapy[79]). In addition, the intervention group will receive active tPCS stimulation while the control group will receive sham tPCS stimulation, during the period of the trial (20 sessions over one month), this will ensure that any estimated treatment differences between groups is unlikely due to placebo effects. All doctors performing pre-post treatment evaluation will be blinded to the allocation of participants to the two groups, participants and their accompany caretakers will also be blinded, thus it will be a double-blind design. The trial recruitment and execution will take place in eight different sites in the Guangdong province in China, to avoid site-specific bias and to verify the feasibility of tPCS in varying clinical environments with patients from different socio-economic backgrounds. The double blinding, sham-controlled, multi-site design will ensure minimal bias to be introduced that may exert confounding effects to the study results.

2 Hypothesis and Objectives

2.1 Hypothesis

tPCS, in addition to standard behavioural therapy, will improve social functioning compared to standard therapy alone in children with ASD aged 3 to 14 years old. We also hypothesize that tPCS will improve sleep disorders in children with ASD aged 3 to 14 years old when compared to standard behavioural therapy alone.

2.2 Primary Objective and Outcome measures

The primary objective is to evaluate the effectiveness of tPCS in addition to standard therapy in improving social functioning in children with ASD, aged 3 to 14 years old. The primary outcome measure will be the change in mean scores as measured by the Autism Treatment Evaluation Checklist (ATEC), for both ATEC total and subdomain scores, at post-treatment compared to baseline. The secondary outcome measure will be the change in mean scores as measured by the Autism Behaviour Checklist (ABC), for both ABC total and subdomain scores, at post-treatment compared to baseline.

2.3 Secondary Objective and Outcome measures

The secondary objective is to assess the effectiveness of tPCS in addition to standard therapy in improving sleep disorders in children with ASD, aged 3 to 14 years old. This secondary outcome measure will be the change in mean scores of the Children' Sleep Habits Questionnaire (CSHQ), for both CSHQ total and subdomain scores ,at post-treatment compared to baseline.

2.4 Potential Risks and Benefits

2.4.1 Potential Risks

tPCS may cause discomfort or tingling sensation to some people. Some people may have skin redness due to allergic reaction to electrode. Few people may complain of headache post stimulation. Otherwise, there is minimal risk in tPCS if there are no contraindication.

2.4.2 Potential Benefit

A significant portion of children with ASD have unmet medical needs that are not adequately addressed by behavioral therapies and certain medications. Use of tPCS may be potentially an effective novel intervention for children with ASD to improve the core symptom of social functioning without potential drug side effects, and with modulating aberrant brain activity such as disrupted E/I balance may offer treatment beyond symptom management. Additionally, tPCS may be effective to treat sleep dysfunctions which are a common comorbidity in ASD , this may reduce caretaker burnout and improve quality of life for the child and the entire family.

3 Study Population

3.1 Number and Nature of Subjects to be Enrolled

340 children with ASD, aged 3 to 14 years old will be enrolled for this study. See section 9.1 for detailed sample calculation.

3.2 Study Settings

Recruitment will be performed onsite at the eight participating sites to achieve the targeted sample size, including:

1) Nanhai Maternity and Children's Hospital of Guangzhou University of Traditional Chinese Medicine,
2) Dongguan Maternal and Child Health Hospital,
3) Zhanjiang Maternal and Child Health Hospital,
4) Luoding Maternal and Child Health Hospital,
5) Meixian District Hospital of Traditional Chinese Medicine,
6) Guangzhou Angel Children's Hospital ,
7) Shenzhen Luogang Maternal and Child Health Hospital and
8) Sichuang Special Needs Education School and Hospital.

3.3 Screening Visits Procedures and Recruitment

Potential participants will be children with ASD coming for clinic visit and receiving inpatient and outpatient pediatric psychiatry, neurology and/or ASD therapy services at the eight sites. The study team doctors on duty will be responsible for identifying potential participants and screened according to the diagnostic, inclusion and exclusion criteria as stated in sections 3.4 to 3.6. Screening per participant will take approximately two hours. If criteria are met, potential participants and their parents /legal guardian who show interest in the study will be given relevant information (standard study participation information sheet) and the consent /assent form (See Appendix 4) in a private room which is comfortable and with privacy to consider carefully. Adequate time will be given to the participant to consider participation. There will be no coercion. Any query will be redirected to the study team doctor on site. Subjects and parents / legal guardians will be informed that participation or not will not affect medical care. If the child has some but not full understanding of the information sheet & consent form, the simplified version (assent form) will be used. However, assent form (between 6 – 14 years old) is omitted if subject is unable to read and understand due to chronic ASD condition. Consent will be taken by anyone from the study team on site. Parents/ legal guardian will sign the consent form in the presence of a witness.

3.4 Diagnostic Criteria

Diagnosis of ASD according to DSM-V (2013) criteria published in the United States[1] and do not have worse than moderate-to-severe intellectual disability according to the Wechsler Intelligence Scale for Children, 4th Edition (WISC-IV)[80]. Diagnosis of ASD will be performed by trained child psychiatrists in each site with at least 5 years of experience. Assessment of intellectual disability will be performed by educational specialists with at least 3 years of experience.

3.5 Inclusion Criteria

A. Subject meets the DSM-V[1] diagnostic criteria for autism spectrum disorder.
B. The subject and parents of the subject gave informed consent to the treatment, agree to commit to the course of treatment and complete the various pre- and post-treatment assessments.
C. Children aged 3-14 years, regardless of gender.
D. Subjects with IQ score > 35 , no worse than moderate intellectual disability, according to WISC (4th edition)

E. No history of epilepsy.

3.6 Exclusion Criteria

A. Those who do not meet the above inclusion criteria;
B. Subjects <3 years or >14 years old;
C. Subjects with IQ score ≤ 35 , according to WISC (4th edition)
D. Subjects with comorbid epilepsy or history of epileptic disorders
E. Subjects with presence of severe psychiatric disorders such as schizophrenia and psychosis, or a family history of schizophrenia and psychosis.
F. Subjects diagnosed with obstructive sleep apnea.
G. Subjects with skull defect or presence of severe scalp infection at the proposed site of stimulation.
H. Subjects with a history of craniotomy, presence of any ferromagnetic metal or implanted medical devices in the head or body, e.g. cochlear implant, pacemaker or defibrillator, history of severe neurological disorders such as brain tumors and intracranial infection
I. Subjects with history of substance abuse or dependence, use of benzodiazepines, neuroleptics and antipsychotic medications including risperidone and haloperidol.
J. Subjects who have current involvement with other forms of non-invasive brain stimulation including transcranial magnetic stimulation or transcranial direct current stimulation.

3.7 Drop-out Criteria

A. Subjects that withdrew midway due to adverse events
B. Subjects where treatment was interrupted for more than 7 days during the study, due to sickness such as acute respiratory tract infection.
C. Subjects who failed to complete the course of treatment due to poor compliance or unexpected changes in parents' demands.
D. Subjects whose circumstances no longer adhere to the inclusion and exclusion criteria midway through the trial, Eg. non-compliance of treatment protocol, subjects who begin a drug or treatment course in the exclusion criteria, without informing the study team.
E. Cases where significant human errors were involved including administration of incorrect treatment dose and not being assessed according to the evaluation standards set in this trial during the pre- and post-treatment assessments.
F. Subjects with significant missing items in the pre- and post-evaluations resulting in incomplete data.

3.8 Provisions for dropout cases and handling of missing data

A. Drop-out definition: As per clause 3.6. Subjects who drop out will not be replaced.
B. Handling of missing data: For subjects who have dropout, the researcher will contact the subjects as soon as possible to ask for the reason for the dropout and make records of the last treatment session. Post-treatment assessments will be completed using actual available data according to best effort basis, where applicable, statistical imputation and other assumptions will be made for Intention-To-Treat analysis. All relevant trial data should be properly kept on file for the dropout cases, which may be required for full analysis.

4 Study Design

The trial design will be a multi-center, double-blind, sham-controlled, parallel group, randomized 1:1 clinical trial involving eight hospitals in China.

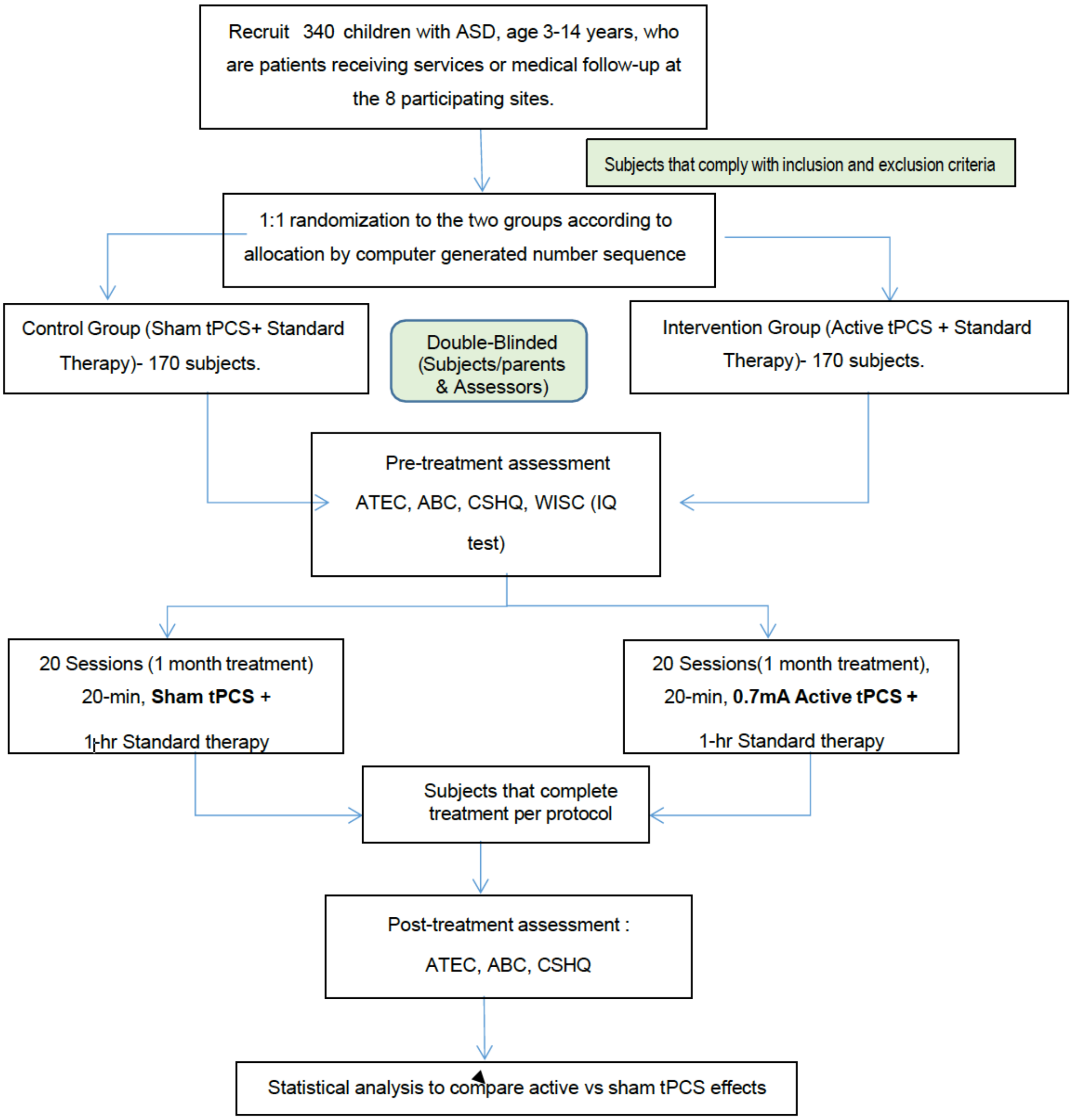

4.1 Randomization - Allocation Sequence Generation

Centralized randomization and allocation will be conducted in accordance with China standards in “Methodology of Clinical Scientific Research of Integrated Traditional Chinese and Western Medicine (2nd edition)”. Recruitment of study subjects will be performed locally at the eight sites, details of recruited subjects will be sent within the same day by Phone/WeChat/Email to an administrator at the main site, Nanhai Maternity and Children’s Hospital, who is uninvolved in the treatment, outcome assessment or final data analysis. The administrator will enter the participant’s details into a secure centralized database. A random allocation sequence will be generated using IBM SPSS27.0 statistical software for 1:1 group division into active-tPCS/ intervention

Group and sham-tPCS/ control Group. 340 random numbers and their corresponding group allocation will be put into 340 opaque, sealed, and identical envelopes. The group allocation will only be marked on the inside of the envelopes.

4.2 Randomization - Allocation Implementation

Subjects who meet the inclusion criteria and have signed consent form will be numbered according to their sequence of the time of visit (from 1 to 340). The administrator will send out the envelope with number corresponding to the number of the subject to the respective hospital of the subject. Upon opening of the envelope, the group allocation is revealed, and the subject is randomized to either the Control (Sham-tPCS) Group or the Intervention (active-tPCS) Group, with 170 subjects in each group.

4.3 Study Visits and Procedures

**Visit to Site**	**Visit 1**	**Visit 2-21**	**Visit 22**
**Timeline**	**Week 0**	**Week 1-4**	**Week 4**
**Procedure**	**Baseline assessment**	**Intervention Period**	**Post-treatment assessment**
Informed Consent[1]	X		
ATEC[2],	X		X
ABC[3],	X		X
CSHQ[4],	X		X
WISC[5]	X		
Urine test	X		X
Real-tPCS[6] + 1 Hour Standard Therapy[7]<br><br>or<br><br>Sham-tPCS[6] + 1 Hour Standard Therapy[7]		X	

[1]Informed Consent

Taken at screening visit, i.e. on the same day as when they are identified as a potential participant. Randomization will be performed as per section 4.1 and 4.2, only after consent is taken (See Appendix 4).

[2]Autism Treatment Evaluation Checklist (ATEC)

ATEC is developed by the Autism Research Institute[81] and designed as a one-page checklist. Comprising 77 items, the ATEC has been utilized to assess the efficacy of interventions by measuring changes in response to various treatment modalities for children with ASD[82]. The ATEC consists of four subscales totaling 77 items:

- Scale I: Speech, Language, and Communication (14 items, with scores ranging from 0 to 28)
- Scale II: Sociability (20 items, with scores ranging from 0 to 40)
- Scale III: Sensory and Cognitive Awareness (18 items, with scores ranging from 0 to 36)
- Scale IV: Health, Physical Condition, and Behavior (25 items, with scores ranging from 0 to 75)

Each category within the ATEC utilizes a four-point rating scale to gauge the severity of issues, ranging from 0 (indicating no problem) to 3 (indicating a serious problem). The ATEC generates a total score ranging from 0 to 179, with higher scores indicating a greater degree of reported activity limitations[82]. This comprehensive assessment tool enables researchers and clinicians to gain insight into the challenges faced by individuals with ASD and to monitor changes in symptoms and functioning over time. For this study, the ATEC will be administered twice (pre- and post-tPCS) and carried out by an experienced therapist or doctor who is blinded regarding the subject allocation.

3 Autism Behaviour Checklist (ABC)

The Autism Behavior Checklist[83] is a classic screening tool for autism. Comprising 57 items and organized into five subscales—sensory behavior, social relating, body and object use, language and communication skills, and social and adaptive skills—the checklist assigns scores ranging from 1 to 4 per item, with higher scores indicating more severe symptoms. A total score of <53 is considered a negative screening test, a total score of ≥53 and ≤67 is considered a positive screening test, and a total score of ≥68 can assist in the diagnosis of autism. In this study, the ABC will be administered twice (pre- and post-tPCS) by an experienced therapist or doctor in clinic, who is blinded regarding the subject allocation.

4 Children's Sleep Habits Questionnaire (CSHQ)

The Children's Sleep Habits Questionnaire (CSHQ)[84,85] is a retrospective assessment tool to investigate sleep patterns in young children consisting of 45 items designed for parents to report on their child's sleep behavior. The CSHQ covers various sleep domains, including bedtime behavior and sleep onset; sleep duration; anxiety around sleep; behavior occurring during sleep and night wakings; sleep-disordered breathing; parasomnias; and morning waking/daytime sleepiness. Each item is rated on a three-point scale: "usually" if the behavior occurred five to seven times per week, "sometimes" for two to four times per week, and "rarely" for zero to one time per week. In this study, the CSHQ will be administered twice (pre- and post-tPCS) by experienced therapists who is blinded regarding the subject allocation through interviewing parents or caregivers in clinic.

5 Wechsler Intelligence Scale for Children, 4th Edition (WISC-IV)

Performed at screening visit, i.e. on the same day as when they are identified as a potential participant, those with IQ score of ≤35 is defined as having severe cognitive impairment and will be excluded from the study. The Wechsler Intelligence Scale for Children, 4th Edition (WISC- IV)[80], is designed for use with children aged 6 to 16 years and 11 months. It provides an assessment of cognitive abilities in children within this age range, helping to identify strengths and weaknesses in various domains of intellectual functioning. An IQ score of <70 is defined as having intellectual disability.

6 Transcranial Pulsed Current Stimulation (tPCS)

- Real-tPCS for the intervention group - 20min/ session of tPCS will be performed at the participating site by a trained therapist or doctor, Monday to Friday for 20 sessions over 4 weeks in the study period.
- Sham-tPCS for the control group – everything the same as active-tPCS, except there is current output for only the first 10s of stimulation and the output will automatically zeroizes for the remaining time of the 20-min session.

- The same therapist/ doctor will not take part in patient assessments.
- Parents / caregivers of both groups will be asked to fill out the adverse event log after each session.

7 Standard Behavioural Therapy

Standard therapy comprises of 1-hour of one of the following therapy programs - Applied Behaviour Analysis (ABA)[76], structured learning[77], play-based therapy [78] and speech therapy[79]. Standard therapy is performed by experienced therapists with at least five-year experience.

4.4 Discontinuation

Study intervention will be discontinued if subject is unable to tolerate the tPCS or standard behavioral therapy as well as if drop-out criteria is met as per Section 3.7. Subjects may withdraw voluntarily from participation in the study at any time due to any reason. If withdrawal occurs, subjects will be asked to complete a form to state the reason for withdrawal.

5 Trial Material

5.1 Trial Product

The tPCS device that will be used is the Multi-channel Pulsed current stimulator, model YQ-D1111, manufactured by YIQI Biotechnology Co. Ltd in China. The device has 11-channels, set at maximum current amplitude of 2mA. Each participant will only use one channel for scalp stimulation. The device can accommodate up to maximum 11 participants at a time in hospital setting. Both the active-tPCS and sham-tPCS devices will be this same model and look identical. The sham device will output current only for the for the first 10 seconds, then zeroized for the remainder session but the light on screen will remain throughout the treatment session to give the impression that stimulation is ongoing.

5.2 Trial Device Characteristics):

- Range of Current Amplitude (all 11 channels): 0.00 mA – max 2mA
- Waveform: Unilateral monophasic square wave
- Frequency: 400Hz
- Pulse width: 140μs

5.3 Electrode materials

Circular silver-fiber cotton electrodes, 4cm-diameter, 12.56cm^2 surface area. Secure to scalp using Velcro head strap (See Figure 2 below)

Figure 2. 4cm-diameter, 12.56cm^2 circular silver-fiber cotton electrode and black Velcro head strap

6 Treatment

6.1 Rationale for Selection of Dose

Based on safety recommendations of tES in children, reduced current intensities (~1 mA) are often used (Krishnan et al., 2015). In this trial, 0.7mA will be the dose for the intervention group, 20 min duration per session. In the active-tPCS group, there was a 10-second ramp-up to 0.7 mA, maintained for the session, and a 10-second ramp-down at the end. In the sham group, there will be a 10-second ramp-up to 0.7 mA followed immediately by a 10-second ramp-down to zero for the remainder of the session.

6.2 Treatment Procedure

Treatment in the current trial will be performed over a one-month period, consisting of the following:

6.2.1 One day (approximately 3 hours) of baseline assessment of ATEC, ABC, CSHQ and WISC, performed by qualified occupational therapists and child psychologists, through clinical observation and parent interviews.

6.2.2 tPCS: Twenty consecutive sessions of active-tPCS in the intervention group or sham-tPCS in the control group, 20-min per session , once a day, 5 times a week (Monday to Friday), over 4 weeks. tPCS will be administered by a trained therapist or doctor in clinic. The same therapist/ doctor will not take part in patient assessments. The current amplitude will be at 0.7mA for active stimulation in the intervention group, with 10 seconds ramp up at the start and 10 seconds ramp down at the end of the session. For sham stimulation in the control group, there will be an output current for the first 10 seconds and then the stimulation intensity will recede to 0, but the power indicator on the front of the device will continue to light up just like in active stimulation, even after the current is discontinued, to allow for masked stimulation.

tPCS Pre-treatment procedures: Subjects receiving active or sham tPCS treatment will first be fitted with a Velcro head strap that is used to firmly secure the anode and cathode circular silver-fiber cotton electrodes to the scalp according to the trial montage i.e. Cathode on the left dorsolateral pre-frontal cortex (L-dLPFC) and Anode on the right cerebellar hemisphere. The electrodes are thoroughly pre-soaked with 0.9%NaCI solution for 10 mins before application.

tPCS Montage (Electrode Placement): See Figure 3. During both the active and sham tPCS treatment, the Anode electrode will be placed on the right cerebellar hemisphere, 1 cm below and 4 cm to the right of the inion (Red), the Cathode electrode will be placed on the left dorsolateral prefrontal cortex, corresponding to the F3 position of the EEG10-20 electrode placement system (White), located using the Beam-F3 method[86]. The electrodes will be secured to the scalp using the pre-fitted Velcro head strap.

Figure 3. Electrode placement for tPCS (Active and Sham)

6.2.3 Concomitant therapy:

After tPCS, participants in both active and sham-tPCS groups will be given standard behavioural therapy over 4 weeks in the study period. There will be a 1-hour therapy session in addition to daily active or sham tPCS. Standard behavioural therapy includes Applied Behaviour Analysis[76], structured education[77], play-based therapy[78] and speech therapy[79]. Each of the four therapies will be administered five times per subject, totaling 20 hours therapy per subject. Standard therapy is performed by experienced therapists with at least five-year experience.

6.2.4 One day (approximately 3 hours) of post-treatment assessment of ATEC, ABC and CSHQ, performed by qualified occupational therapists and child psychologists, through clinical observation and parent interviews.

6.3 Specific treatment adherence requirements/strategies

In this study , the tPCS intervention will be provided to participants free-of-charge in the eight hospital sites. The participants will not receive any monetary payments or gifts for their participation in the study.

6.4 Blinding

The study is double-blinded, both the study subjects (including their parents /caretakers) and outcome evaluators are blinded and will not know about the group allocation. The devices used in the active and sham tPCS groups will be identical, each hospital involved in this study will allocate two separate treatment rooms to place the active-tPCS and sham-tPCS devices. The two rooms will be reasonably separated in different levels of the building so that there will be no chance of communication between the two groups. Participants and accompanying parents/guardians will be asked to return to the same treatment room for all 20 recurring daily treatments. All subjects will be naïve users of tPCS, they will not be aware of the difference between active and sham stimulation condition and will be informed by the treating doctor that the sensation felt is a typical tPCS sensation, effectively blinding subjects to the different treatment conditions. Given that tPCS is non-invasive and effects of tPCS are temporary, there will be no emergency unblinding in this trial. Should SAEs occur, safety handling will follow procedures as stated under Section 7.

6.4.1 **Outcome Assessors**

Two qualified neurodevelopmental specialist doctors (child psychiatrists and/or pediatric neurologists) from each of the eight participating sites with more than five years of experience will be responsible for baseline and post-

treatment evaluation. All medical personnel participating in the outcome assessment process will be required to undergo mandatory 2-day refresher training on standardized assessments of ATEC, ABC, and CSHQ to reduce rater bias. Training will take place before study commencement in person in each of the site. The outcome assessors are forbidden to be involved in the subject randomized allocation to the two groups and will be blinded. The outcome assessors will also not participate in the treatment of the subjects.

6.4.2 **Subjects**

Subjects in the intervention group will be given active-tPCS stimulation and subjects in the Control group will be given sham-tPCS stimulation. The subjects and their parents / caretakers will be blinded according to Section 6.4.

7 Safety Measurements

7.1 Definitions

An adverse event (AE) is any untoward medical occurrence in a clinical investigation subject who is administered a medicinal product or investigational product, and which does not necessarily have a causal relationship with this treatment. An adverse event (AE) can therefore be any unfavorable and unintended sign (including an abnormal laboratory finding), symptom, or disease temporally associated with the use of a medicinal (investigational) product, whether or not related to the medicinal (investigational) product. A serious adverse event (SAE) is any untoward medical occurrence that at any dose:

- results in death
- is life-threatening
- requires inpatient hospitalization or prolongation of existing hospitalization
- results in persistent or significant disability/incapacity, or
- is a congenital anomaly/birth defect

7.2 Collection, Recording and Reporting of Serious Adverse Events to Ethics Board

Only related SAEs (definitely/ probably/ possibly) will be reported to the Ethics Board of Nanhai Maternity and Children's Hospital of Guangzhou University of Chinese Medicine (main hospital site) and the Foshan Science and Technology Bureau, Guangdong province. Related means there is a reasonable possibility that the event may have been caused by participation in the clinical trial. The investigator is responsible for informing the Ethics Board after first knowledge that the case qualifies for reporting. Follow-up information will be actively sought and submitted as it becomes available. Related AEs will not be reported to Ethics Board. However, the investigator is responsible to keep record of such AEs cases at the Study Site File.

7.3 Safety Monitoring Plan

All data will be entered into a secure password-protected document in the desktop. Access to the data will only be open to the Principal Investigator and Co-Investigators. Hardcopy of the data forms will be stored in a locked cupboard in the department. All adverse events of tPCS in all participants will also be logged and safety of tPCS will be monitored by the Principal Investigator and Co-Investigators.

7.4 Complaint Handling

If there is any complaint of the tPCS, the Principal Investigator and Co-Investigators will be informed immediately to rectify the issue. If the subject is unable to tolerate the tPCS, the study will be discontinued for the subject.

8 Data Analysis

8.1 Data Quality Assurance

All study team members will be trained in the study-related procedures as delegated to ensure that collected data is reliable and complete. Identifiable patient data will be anonymized. Access to the data will only be open to the Principal Investigator and Co-Investigators. The Principal Investigator and Co-Investigators will ensure adherence with the protocol and accuracy in relation to data entry. Quality of the data will be ensured and monitored every month during the study period, the Principal Investigator and Co-Investigators will maintain essential study documents (protocol and amendments, source documentation, relevant correspondence, and all other supporting documentation) in a confidential manner as required by the approving ethics committee.

8.2 Data Entry and Storage

Data may be first recorded on hard copies of the standardized data forms of ATEC, ABC and CSHQ (*see Appendix 1-3 for data forms*), and subsequently keyed into a secure password-protected document on a shared drive among the eight sites. Data entry and subsequent data quality checks will be performed by two designated personnel at each site, at the end of every month during the study period. Access to the data will only be open to the study investigators. The Principal Investigator and Co-investigators will ensure accuracy of the data entered. Hardcopy of the data forms will be stored in locked cupboards in the respective hospital pediatric departments.

9 Sample Size and Statistical Methods

9.1 Determination of Sample Size

Based on a two-sided paired t-test at 80% power, 5% one-sided alpha and an expected effect size of small (0.20)[35] when using bilateral frontal montage and medium (0.52)[67] when using prefrontal-cerebellar montage, we will use an average of 0.36 effect size to compute the sample size, which leads to 120 per group. Based on an estimated 40% drop-out rate due to potential implications from post- COVID heightened travelling restrictions in Guangdong province, a sample size of 339 is required. We will use 340 as the sample size for recruitment in this study.

9.2 Statistical and Analytical Plans

9.2.1 General Considerations

Statistical analysis will be implemented using the IBM SPSS version 27.0. Before applying any statistical test, the data sets will be tested for a normal distribution with the Shapiro–Wilk test. If the data was found to be normally distributed, then one- way analysis of variance ANOVA and Student's t-test will be used for the comparison between groups. Analysis of Covariance ANCOVA will be used to compare one or more means between groups

while controlling for the effects of one or more covariates (continuous variables that are related to the dependent variable). For comparison with the baseline value, paired t-test or one-way analysis of variance (ANOVA) will be used to compare the differences within the group before and after treatment. If the data was found to be not normally distributed then Kruskal-Wallis test or Mann-Whitney rank-sum test was used for the comparison. In order to correct for multiple comparisons, Bonferroni correction will be used to further validate the significance of each p-value. All statistical tests were conducted using a two-sided test .The α- value was set to 0.05 for all the statistical tests and < 0.01 for Bonferroni correction. Chi-Squared test was used for categorical data, and Mann-Whitney rank sum test was used for ordinal data.

9.2.2 Analysis of dropout cases

The overall dropout rate was compared to the dropout rate due to adverse events using the Chi-Squared test; Cases lost to follow-up and patients who stopped the trial voluntarily should be included in the analysis of dropout cases.

9.2.3 Safety analysis

Intervention is non-invasive, potential side effects are only limited to influences upon the skin where the electrodes are placed. Participant discomfort will be closely monitored. Urine test will be conducted before and after the intervention to check for any unintended metabolic changes. Adverse event monitoring will be reported in the full study population and described in a table. Chi-Square test will be used for any comparison of the incidence rate of adverse events between the two groups.

10 Direct Access to Source Data/ Documents

The Principal Investigator and Co-Investigators will permit study-related monitoring, audits and/or IRB review and regulatory inspection(s), providing direct access to source data/document.

11 Ethical Considerations

This study will be conducted in accordance with the ethical principles that have their origin in the Declaration of Helsinki (2013 edition) and that are consistent with the applicable regulatory requirements of Good Clinical Practice in China. This final Clinical Trial Protocol, including the final version of the Participant Information Sheet and Consent Form, must be approved in writing by the Centralized Research Ethics Committee of the lead center, Nanhai Maternity and Children's Hospital, prior to enrolment of any patient into the study. The principal investigator is responsible for informing the Centralized Research Ethics Committee of any amendments to the protocol or other study-related documents, as per local requirement.

11.1 Confidentiality of Data and Patient Records

All data will be entered into a secure password-protected document in the desktop. Identifiable patient data will be anonymized. Access to the full data will only be open to the Principal Investigator and Co-investigators. Collaborators involved in statistical analysis will be given access to anonymized data on a need-to-know basis. Hardcopy of the data forms will be stored in locked cupboards in the respective hospital pediatric departments.

12 Publications

Following approval by Research Ethics Committee, the study will be registered on Chinese Clinical Trial Registry (www.ChiCTR.org.cn) by the Principal Investigator. Study results will be published in peer-reviewed scientific literature regardless of the trial's outcome. The CONSORT 2010 Statement will be adhered to in publication of study findings to ensure that international standards of authorship and publication are met. Study results, including participant-level dataset and statistical code will be made available on ResMan database managed by the Chinese Clinical Trial Registry.

13 Retention of Trial Document

When the study is completed, the research data will be kept in a secure password-protected document in the desktop at the department. All the documents will be retained for at least 7 years after completion of the research study. The Principal Investigator and Co-Investigators will have access to the data. Regular audits will be done by the Principal Investigator and Co-Investigators to ensure data integrity and accuracy.

14 Funding

This study was funded by NUS Department of Psychological Medicine (R-177-000-100-001/R-177-000- 003-001/ R177000702733), National University of Singapore iHeathtech Other Operating Expenses (A- 0001415-09-00) and Guangzhou Yirui Charitable Foundation, a charitable organization in China that supports special needs children. The grant amount covers research-related costs such as fees for use of assessment scale and procedure-related costs.

References

1. American Psychiatric Association. Diagnostic and Statistical Manual of Mental Disorders. American Psychiatric Association; 2013. doi:10.1176/appi.books.9780890425596
2. Souders MC, Zavodny S, Eriksen W, et al. Sleep in Children with Autism Spectrum Disorder. Curr Psychiatry Rep. 2017;19(6):34. doi:10.1007/s11920-017-0782-x
3. Polimeni MA, Richdale AL, Francis AJP. A survey of sleep problems in autism, Asperger's disorder and typically developing children. Journal of Intellectual Disability Research. 2005;49(4):260-268. doi:10.1111/j.1365-2788.2005.00642.x
4. KRAKOWIAK P, GOODLIN-JONES B, HERTZ-PICCIOTTO I, CROEN LA, HANSEN RL. Sleep problems in children with autism spectrum disorders, developmental delays, and typical development: a population-based study. J Sleep Res. 2008;17(2):197-206. doi:10.1111/j.1365-2869.2008.00650.x
5. Posar A, Visconti P. Sleep Problems in Children with Autism Spectrum Disorder. Pediatr Ann. 2020;49(6). doi:10.3928/19382359-20200511-01
6. Maenner MJ, Warren Z, Williams AR, et al. Prevalence and Characteristics of Autism Spectrum Disorder Among Children Aged 8 Years — Autism and Developmental Disabilities Monitoring Network, 11 Sites, United States, 2020. MMWR Surveillance Summaries. 2023;72(2):1-14. doi:10.15585/mmwr.ss7202a1
7. Anixt JS, Ehrhardt J, Duncan A. Evidence-Based Interventions in Autism. Pediatr Clin North Am. 2024;71(2):199-221. doi:10.1016/j.pcl.2024.01.001
8. Persico AM, Ricciardello A, Lamberti M, et al. The pediatric psychopharmacology of autism spectrum disorder:

A systematic review - Part I: The past and the present. Prog Neuropsychopharmacol Biol Psychiatry. 2021;110:110326. doi:10.1016/j.pnpbp.2021.110326

9. Fuller EA, Kaiser AP. The Effects of Early Intervention on Social Communication Outcomes for Children with Autism Spectrum Disorder: A Meta-analysis. J Autism Dev Disord. 2020;50(5):1683-1700. doi:10.1007/s10803-019-03927-z
10. Zhou MS, Nasir M, Farhat LC, Kook M, Artukoglu BB, Bloch MH. Meta-analysis: Pharmacologic Treatment of Restricted and Repetitive Behaviors in Autism Spectrum Disorders. J Am Acad Child Adolesc Psychiatry. 2021;60(1):35-45. doi:10.1016/j.jaac.2020.03.007
11. Hock R, Kinsman A, Ortaglia A. Examining treatment adherence among parents of children with autism spectrum disorder. Disabil Health J. 2015;8(3):407-413. doi:10.1016/j.dhjo.2014.10.005
12. Devlin B, Scherer SW. Genetic architecture in autism spectrum disorder. Curr Opin Genet Dev. 2012;22(3):229-237. doi:10.1016/j.gde.2012.03.002
13. Grove J, Ripke S, Als TD, et al. Identification of common genetic risk variants for autism spectrum disorder. Nat Genet. 2019;51(3):431-444. doi:10.1038/s41588-019-0344-8
14. Hulbert SW, Jiang YH. Monogenic mouse models of autism spectrum disorders: Common mechanisms and missing links. Neuroscience. 2016;321:3-23. doi:10.1016/j.neuroscience.2015.12.040
15. Karimi P, Kamali E, Mousavi S, Karahmadi M. Environmental factors influencing the risk of autism. Journal of Research in Medical Sciences. 2017;22(1):27. doi:10.4103/1735-1995.200272
16. Minshew NJ, Williams DL. The New Neurobiology of Autism. Arch Neurol. 2007;64(7):945. doi:10.1001/archneur.64.7.945
17. Just MA, Cherkassky VL, Keller TA, Kana RK, Minshew NJ. Functional and Anatomical Cortical Underconnectivity in Autism: Evidence from an fMRI Study of an Executive Function Task and Corpus Callosum Morphometry. Cerebral Cortex. 2007;17(4):951-961. doi:10.1093/cercor/bhl006
18. Trakoshis S, Martínez-Cañada P, Rocchi F, et al. Intrinsic excitation-inhibition imbalance affects medial prefrontal cortex differently in autistic men versus women. Elife. 2020;9. doi:10.7554/eLife.55684
19. D’Mello AM, Stoodley CJ. Cerebro-cerebellar circuits in autism spectrum disorder. Front Neurosci. 2015;9. doi:10.3389/fnins.2015.00408
20. Verly M, Verhoeven J, Zink I, et al. Altered functional connectivity of the language network in ASD: Role of classical language areas and cerebellum. Neuroimage Clin. 2014;4:374-382. doi:10.1016/j.nicl.2014.01.008
21. Becker EBE, Stoodley CJ. Autism Spectrum Disorder and the Cerebellum. In: ; 2013:1-34. doi:10.1016/B978-0-12-418700-9.00001-0
22. Stoodley CJ, Tsai PT. Adaptive Prediction for Social Contexts: The Cerebellar Contribution to Typical and Atypical Social Behaviors. Annu Rev Neurosci. 2021;44(1):475-493. doi:10.1146/annurev-neuro-100120-092143
23. Kayarian FB, Jannati A, Rotenberg A, Santarnecchi E. Targeting Gamma-Related Pathophysiology in Autism Spectrum Disorder Using Transcranial Electrical Stimulation: Opportunities and Challenges. Autism Research. 2020;13(7):1051-1071. doi:10.1002/aur.2312
24. J L R Rubenstein, M M Merzenich. Model of autism: increased ratio of excitation/inhibition in key neural systems.
25. Manyukhina VO, Prokofyev AO, Galuta IA, et al. Globally elevated excitation–inhibition ratio in children with autism spectrum disorder and below-average intelligence. Mol Autism. 2022;13(1):20. doi:10.1186/s13229-022-

00498-2

26. Nelson SB, Valakh V. Excitatory/Inhibitory Balance and Circuit Homeostasis in Autism Spectrum Disorders. Neuron. 2015;87(4):684-698. doi:10.1016/j.neuron.2015.07.033
27. Bandeira ID, Guimarães RSQ, Jagersbacher JG, et al. Transcranial Direct Current Stimulation in Children and Adolescents With Attention-Deficit/Hyperactivity Disorder (ADHD). J Child Neurol. 2016;31(7):918-924. doi:10.1177/0883073816630083
28. Prehn-Kristensen A, Munz M, Göder R, et al. Transcranial Oscillatory Direct Current Stimulation During Sleep Improves Declarative Memory Consolidation in Children With Attention-deficit/hyperactivity Disorder to a Level Comparable to Healthy Controls. Brain Stimul. 2014;7(6):793-799. doi:10.1016/j.brs.2014.07.036
29. Hamilton A, Wakely L, Marquez J. Transcranial Direct-Current Stimulation on Motor Function in Pediatric Cerebral Palsy: A Systematic Review. Pediatric Physical Therapy. 2018;30(4):291-301. doi:10.1097/PEP.0000000000000535
30. Liu Z, Dong S, Zhong S, et al. The effect of combined transcranial pulsed current stimulation and transcutaneous electrical nerve stimulation on lower limb spasticity in children with spastic cerebral palsy: a randomized and controlled clinical study. BMC Pediatr. 2021;21(1):141. doi:10.1186/s12887-021-02615-1
31. Costanzo F, Rossi S, Varuzza C, Varvara P, Vicari S, Menghini D. Long-lasting improvement following tDCS treatment combined with a training for reading in children and adolescents with dyslexia. Neuropsychologia. 2019;130:38-43. doi:10.1016/j.neuropsychologia.2018.03.016
32. Lazzaro G, Costanzo F, Varuzza C, et al. Individual Differences Modulate the Effects of tDCS on Reading in Children and Adolescents with Dyslexia. Scientific Studies of Reading. 2021;25(6):470-485. doi:10.1080/10888438.2020.1842413
33. Amatachaya A, Auvichayapat N, Patjanasoontorn N, et al. Effect of Anodal Transcranial Direct Current Stimulation on Autism: A Randomized Double-Blind Crossover Trial. Behavioural Neurology. 2014;2014:1-7. doi:10.1155/2014/173073
34. García-González S, Lugo-Marín J, Setien-Ramos I, et al. Transcranial direct current stimulation in Autism Spectrum Disorder: A systematic review and meta-analysis. European Neuropsychopharmacology. 2021;48:89-109. doi:10.1016/j.euroneuro.2021.02.017
35. Hadoush H, Nazzal M, Almasri NA, Khalil H, Alafeef M. Therapeutic Effects of Bilateral Anodal Transcranial Direct Current Stimulation on Prefrontal and Motor Cortical Areas in Children with Autism Spectrum Disorders: A Pilot Study. Autism Research. 2020;13(5):828-836. doi:10.1002/aur.2290
36. Mahmoodifar E, Sotoodeh MS. Combined Transcranial Direct Current Stimulation and Selective Motor Training Enhances Balance in Children With Autism Spectrum Disorder. Percept Mot Skills. 2020;127(1):113-125. doi:10.1177/0031512519888072
37. Toscano E, Sanges V, Riccio MP, Bravaccio C, de Bartolomeis A, D'Urso G. Fronto-cerebellar tDCS in children with Autism Spectrum Disorder. Encephale. 2019;45:S79-S80. doi:10.1016/j.encep.2019.04.040
38. Krause B, Cohen Kadosh R. Can transcranial electrical stimulation improve learning difficulties in atypical brain development? A future possibility for cognitive training. Dev Cogn Neurosci. 2013;6:176-194. doi:10.1016/j.dcn.2013.04.001
39. Ma Z, Du X, Wang F, et al. Cortical Plasticity Induced by Anodal Transcranial Pulsed Current Stimulation Investigated by Combining Two-Photon Imaging and Electrophysiological Recording. Front Cell Neurosci. 2019;13. doi:10.3389/fncel.2019.00400

40. Thibaut A, Russo C, Hurtado-Puerto AM, et al. Effects of Transcranial Direct Current Stimulation, Transcranial Pulsed Current Stimulation, and Their Combination on Brain Oscillations in Patients with Chronic Visceral Pain: A Pilot Crossover Randomized Controlled Study. Front Neurol. 2017;8. doi:10.3389/fneur.2017.00576
41. Nitsche MA, Paulus W. Excitability changes induced in the human motor cortex by weak transcranial direct current stimulation. J Physiol. 2000;527(3):633-639. doi:10.1111/j.1469-7793.2000.t01-1-00633.x
42. Guleyupoglu B, Schestatsky P, Edwards D, Fregni F, Bikson M. Classification of methods in transcranial Electrical Stimulation (tES) and evolving strategy from historical approaches to contemporary innovations. J Neurosci Methods. 2013;219(2):297-311. doi:10.1016/j.jneumeth.2013.07.016
43. Alon G, Syron SC, Smith G V. Is Transcranial Electrical Stimulation (TCES) a Safe Intervention for Children with Cerebral Palsy? Meath Drive; 1144.
44. Barra A, Rosenfelder M, Mortaheb S, et al. Transcranial Pulsed-Current Stimulation versus Transcranial Direct Current Stimulation in Patients with Disorders of Consciousness: A Pilot, Sham-Controlled Cross-Over Double-Blind Study. Brain Sci. 2022;12(4):429. doi:10.3390/brainsci12040429
45. Castillo Saavedra L, Morales-Quezada L, Doruk D, et al. QEEG indexed frontal connectivity effects of transcranial pulsed current stimulation (tPCS): A sham-controlled mechanistic trial. Neurosci Lett. 2014;577:61-65. doi:10.1016/j.neulet.2014.06.021
46. Morales-Quezada L, Leite J, Carvalho S, Castillo-Saavedra L, Cosmo C, Fregni F. Behavioral effects of transcranial pulsed current stimulation (tPCS): Speed-accuracy tradeoff in attention switching task. Neurosci Res. 2016;109:48-53. doi:10.1016/j.neures.2016.01.009
47. Morales-Quezada L, Saavedra LC, Rozisky J, Hadlington L, Fregni F. Intensity-dependent effects of transcranial pulsed current stimulation on interhemispheric connectivity. Neuroreport. 2014;25(13):1054-1058. doi:10.1097/WNR.0000000000000228
48. Datta A, Dmochowski JP, Guleyupoglu B, Bikson M, Fregni F. Cranial electrotherapy stimulation and transcranial pulsed current stimulation: A computer based high-resolution modeling study. Neuroimage. 2013;65:280-287. doi:10.1016/j.neuroimage.2012.09.062
49. Dissanayaka T, Zoghi M, Farrell M, Egan G, Jaberzadeh S. The effects of a single-session cathodal transcranial pulsed current stimulation on corticospinal excitability: A randomized sham-controlled double-blinded study. European Journal of Neuroscience. 2020;52(12):4908-4922. doi:10.1111/ejn.14916
50. Dissanayaka T, Zoghi M, Hill AT, Farrell M, Egan G, Jaberzadeh S. The Effect of Transcranial Pulsed Current Stimulation at 4 and 75 Hz on Electroencephalography Theta and High Gamma Band Power: A Pilot Study. Brain Connect. 2020;10(9):520-531. doi:10.1089/brain.2020.0756
51. Jaberzadeh S, Bastani A, Zoghi M. Anodal transcranial pulsed current stimulation: A novel technique to enhance corticospinal excitability. Clinical Neurophysiology. 2014;125(2):344-351. doi:10.1016/j.clinph.2013.08.025
52. Jaberzadeh S, Bastani A, Zoghi M, Morgan P, Fitzgerald PB. Anodal Transcranial Pulsed Current Stimulation: The Effects of Pulse Duration on Corticospinal Excitability. PLoS One. 2015;10(7):e0131779. doi:10.1371/journal.pone.0131779
53. Ruhnau P, Rufener KS, Heinze HJ, Zaehle T. Pulsed transcranial electric brain stimulation enhances speech comprehension. Brain Stimul. 2020;13(5):1402-1411. doi:10.1016/j.brs.2020.07.011
54. Thibaut A, Russo C, Morales-Quezada L, et al. Neural signature of tDCS, tPCS and their combination: Comparing the effects on neural plasticity. Neurosci Lett. 2017;637:207-214. doi:10.1016/j.neulet.2016.10.026
55. Vasquez AC, Thibaut A, Morales-Quezada L, Leite J, Fregni F. Patterns of brain oscillations across different

electrode montages in transcranial pulsed current stimulation. Neuroreport. 2017;28(8):421-425. doi:10.1097/WNR.0000000000000772

56. Vasquez A, Malavera A, Doruk D, et al. Duration Dependent Effects of Transcranial Pulsed Current Stimulation (tPCS) Indexed by Electroencephalography. Neuromodulation: Technology at the Neural Interface. 2016;19(7):679-688. doi:10.1111/ner.12457
57. Jaberzadeh S, Zoghi M. Exploring sensory, motor, and pain responses as potential side or therapeutic effects of sub-2 mA, 400 Hz transcranial pulsed current stimulation. PLoS One. 2023;18(12):e0290137. doi:10.1371/journal.pone.0290137
58. Sours C, Alon G, Roys S, Gullapalli RP. Modulation of Resting State Functional Connectivity of the Motor Network by Transcranial Pulsed Current Stimulation. Brain Connect. 2014;4(3):157-165. doi:10.1089/brain.2013.0196
59. Maximo JO, Cadena EJ, Kana RK. The Implications of Brain Connectivity in the Neuropsychology of Autism. Neuropsychol Rev. 2014;24(1):16-31. doi:10.1007/s11065-014-9250-0
60. Zhao H, Mao X, Zhu C, et al. GABAergic System Dysfunction in Autism Spectrum Disorders. Front Cell Dev Biol. 2022;9. doi:10.3389/fcell.2021.781327
61. Buzsáki G, Wang XJ. Mechanisms of Gamma Oscillations. Annu Rev Neurosci. 2012;35(1):203-225. doi:10.1146/annurev-neuro-062111-150444
62. Morales-Quezada L, Castillo-Saavedra L, Cosmo C, et al. Optimal random frequency range in transcranial pulsed current stimulation indexed by quantitative electroencephalography. Neuroreport. 2015;26(13):747-752. doi:10.1097/WNR.0000000000000415
63. Kelly E, Meng F, Fujita H, et al. Regulation of autism-relevant behaviors by cerebellar–prefrontal cortical circuits. Nat Neurosci. 2020;23(9):1102-1110. doi:10.1038/s41593-020-0665-z
64. Auvichayapat N, Patjanasoontorn N, Phuttharak W, et al. Brain Metabolite Changes After Anodal Transcranial Direct Current Stimulation in Autism Spectrum Disorder. Front Mol Neurosci. 2020;13. doi:10.3389/fnmol.2020.00070
65. Gómez L, Vidal B, Maragoto C, et al. Non-invasive brain stimulation for children with autism spectrum disorders: A short-term outcome study. Behavioral Sciences. 2017;7(3). doi:10.3390/bs7030063
66. D’Urso G, Bruzzese D, Ferrucci R, et al. Transcranial direct current stimulation for hyperactivity and noncompliance in autistic disorder. World Journal of Biological Psychiatry. 2015;16(5):361-366. doi:10.3109/15622975.2015.1014411
67. D’Urso G, Toscano E, Sanges V, et al. Cerebellar Transcranial Direct Current Stimulation in Children with Autism Spectrum Disorder: A Pilot Study on Efficacy, Feasibility, Safety, and Unexpected Outcomes in Tic Disorder and Epilepsy. J Clin Med. 2021;11(1):143. doi:10.3390/jcm11010143
68. Alon G, Yungher DA, Shulman LM, Rogers MW. Safety and Immediate Effect of Noninvasive Transcranial Pulsed Current Stimulation on Gait and Balance in Parkinson Disease. Neurorehabil Neural Repair. 2012;26(9):1089-1095. doi:10.1177/1545968312448233
69. Antal A, Alekseichuk I, Bikson M, et al. Low intensity transcranial electric stimulation: Safety, ethical, legal regulatory and application guidelines. Clinical Neurophysiology. 2017;128(9):1774-1809. doi:10.1016/j.clinph.2017.06.001
70. Krishnan C, Santos L, Peterson MD, Ehinger M. Safety of Noninvasive Brain Stimulation in Children and Adolescents. Brain Stimul. 2015;8(1):76-87. doi:10.1016/j.brs.2014.10.012

71. Arul-Anandam AP, Loo C, Mitchell P. Induction of Hypomanic Episode With Transcranial Direct Current Stimulation. J ECT. 2010;26(1):68-69. doi:10.1097/YCT.0b013e3181a744bf
72. Baccaro A, Brunoni AR, Bensenor IM, Fregni F. Hypomanic episode in unipolar depression during transcranial direct current stimulation. Acta Neuropsychiatr. 2010;22(6):316-318. doi:10.1111/j.1601-5215.2010.00495.x
73. Brunoni AR, Valiengo L, Zanao T, de Oliveira JF, Bensenor IM, Fregni F. Manic Psychosis After Sertraline and Transcranial Direct-Current Stimulation. J Neuropsychiatry Clin Neurosci. 2011;23(3):E4-E5. doi:10.1176/jnp.23.3.jnpe4
74. Nitsche MA, Lampe C, Antal A, et al. Dopaminergic modulation of long-lasting direct current-induced cortical excitability changes in the human motor cortex. European Journal of Neuroscience. 2006;23(6):1651-1657. doi:10.1111/j.1460-9568.2006.04676.x
75. Brunoni AR, Ferrucci R, Bortolomasi M, et al. Interactions between transcranial direct current stimulation (tDCS) and pharmacological interventions in the Major Depressive Episode: Findings from a naturalistic study. European Psychiatry. 2013;28(6):356-361. doi:10.1016/j.eurpsy.2012.09.001
76. Miltenberger RG, Valbuena D, Sanchez S. Applied behavior analysis. In: Handbook of Cognitive Behavioral Therapy: Overview and Approaches (Vol. 1). American Psychological Association; 2021:637-671. doi:10.1037/0000218-022
77. Suprihatin S, Tarjiah I. Evaluating the Outcome of Structured Teaching Intervention for Children with Autism. In: Proceedings of the 5th International Conference on Education and Technology (ICET 2019). Atlantis Press; 2019. doi:10.2991/icet-19.2019.72
78. Dougherty J, Ray D. Differential impact of play therapy on developmental levels of children. International Journal of Play Therapy. 2007;16(1):2-19. doi:10.1037/1555-6824.16.1.2
79. Binns A V, Smyth R, Andres A, Lam J, Oram Cardy J. Looking back and moving forward: A scoping review of research on preschool autism interventions in the field of speech-language pathology. Autism Dev Lang Impair. 2021;6:239694152110331. doi:10.1177/23969415211033171
80. Wechsler D. Wechsler Intelligence Scale for Children, Fourth Edition (WISC-IV).; 2003.
81. Rimland B, & ESM (1999). Autism Treatment Evaluation Checklist. [Database record]. APA PsycTests.
82. Magiati I, Moss J, Yates R, Charman T, Howlin P. Is the Autism Treatment Evaluation Checklist a useful tool for monitoring progress in children with autism spectrum disorders? Journal of Intellectual Disability Research. 2011;55(3):302-312. doi:10.1111/j.1365-2788.2010.01359.x
83. Krug DA, Arick J, Almond P. BEHAVIOR CHECKLIST FOR IDENTIFYING SEVERELY HANDICAPPED INDIVIDUALS WITH HIGH LEVELS OF AUTISTIC BEHAVIOR. Journal of Child Psychology and Psychiatry. 1980;21(3):221-229. doi:10.1111/j.1469-7610.1980.tb01797.x
84. Owens J, Maxim R, McGuinn M, Nobile C, Msall M, Alario A. Television-viewing Habits and Sleep Disturbance in School Children. Pediatrics. 1999;104(3):e27-e27. doi:10.1542/peds.104.3.e27
85. Seifer R. Parental Psychopathology and Children's Sleep. In: Sleep and Development. Oxford University Press; 2011:79-98. doi:10.1093/acprof:oso/9780195395754.003.0004
86. Fabregat-Sanjuan A, Pàmies-Vilà R, Pascual-Rubio V. Evaluation of the Beam-F3 method for locating the F3 position from the 10–20 international system. Brain Stimul. 2022;15(4):1011-1012. doi:10.1016/j.brs.2022.07.002

Appendix 1 – Data Form (ATEC)

Autism Treatment Evaluation Checklist (ATEC)

This form is intended to measure the effects of treatment.

[illegible]

Name of Child ____________________ ____________________ ☐ Male Age __________

Last First ☐ Female Date of Birth __________

Form completed by: ____________________ Relationship: ______________ Today's Date __________

Please circle the letters to indicate how true each phrase is:

I. Speech/Language/Communication: *[N] Not true [S] Somewhat true [V] Very true*

N S V 1. Knows own name
N S V 2. Responds to 'No' or 'Stop'
N S V 3. Can follow some commands
N S V 4. Can use one word at a time (No!, Eat, Water, etc.)
N S V 5. Can use 2 words at a time (Don't want, Go home)
N S V 6. Can use 3 words at a time (Want more milk)
N S V 7. Knows 10 or more words
N S V 8. Can use sentences with 4 or more words
N S V 9. Explains what he/she wants
N S V 10. Asks meaningful questions
N S V 11. Speech tends to be meaningful/ relevant
N S V 12. Often uses several successive sentences
N S V 13. Carries on fairly good conversation
N S V 14. Has normal ability to com- municate for his/her age

II. Sociability: *[N] Not descriptive [S] Somewhat descriptive [V] Very descriptive*

N S V 1. Seems to be in a shell – you cannot reach him/her
N S V 2. Ignores other people
N S V 3. Pays little or no attention when addressed
N S V 4. Uncooperative and resistant
N S V 5. No eye contact
N S V 6. Prefers to be left alone
N S V 7. Shows no affection
N S V 8. Fails to greet parents
N S V 9. Avoids contact with others
N S V 10. Does not imitate
N S V 11. Dislikes being held/cuddled
N S V 12. Does not share or show
N S V 13. Does not wave 'bye bye'
N S V 14. Disagreeable/not compliant
N S V 15. Temper tantrums
N S V 16. Lacks friends/companions
N S V 17. Rarely smiles
N S V 18. Insensitive to other's feelings
N S V 19. Indifferent to being liked
N S V 20. Indifferent if parent(s) leave

ID. Sensory/Cognitive Awareness: *[NJ Not descriptive [SJ Somewhat descriptive [VJ Very descriptive*

N S V, 1. Responds to own name
N S V 2. Responds to praise
N S V 3. Looks at people and animals
N S V 4. Looks at pictures (and T.V.)
N S V 5. Does drawing, coloring, art
N S V 6. Plays with toys appropriately
N S V 7. Appropriate facial expression
N S V 8. Understands stories on T.V.
N S V 9. Understands explanations
N S V 10. Aware of environment
N S V 11. Aware of danger
N S V 12. Shows imagination
N S V 13. Initiates activities
N S V 14. Dresses self
N S V 15. Curious, interested
N S V 16. Venturesome - explores
N S V 17. "Tuned in" - Not spacey
N S V 18. Looks where others are looking

IV. Health/Physical/Behavior: *Use this code:* *[NJ Not a Problem [MI] Minor Problem* ***[MO]*** *Moderate Problem [SJ Serious Problem*

N MI MO S 1. Bed-wetting
N MI MO S 2. Wets pants/diapers
N MI MO S 3. Soils pants/diapers
N MI MO S 4. Diarrhea
N MI MO S 5. Constipation
N MI MO S 6. Sleep problems
N MI MO S 7. Eats too much/too little
N MI MO S 8. Extremely limited diet
N MI MO S 9. Hyperactive
N MI MO S 10. Lethargic
N MI MO S 11. Hits or injures self
N MI MO S 12. Hits or injures others
N MI MO S 13. Destructive
N MI MO S 14. Sound-sensitive
N MI MO S 15. Anxious/fearful
N MI MO S 16. Unhappy/crying
N MI MO S 17. Seizures
N MI MO S 18. Obsessive speech
N MI MO S 19. Rigid routines
N MI MO S 20. Shouts or screams
N MI MO S 21. Demands sameness
N MI MO S 22. Often agitated
N MI MO S 23. Not sensitive to pain
N MI MO S 24. "Hooked" or fixated on certain objects/topics
N MI MO S 25. Repetitive movements (stimming, rocking, etc.)

Appendix 2 – Data Form (ABC)

Autism Behaviour Checklist

Child's Name: ______Gender: ___Date of Birth: ______ Year ____ Month ____ Day

Relationship to the Child: ____________Contact Information: ______

Date of Form Completion: ______ Year ____ Month ____ Day

(Note: The person filling out the form should be the child’s parents or someone who has lived with the child for more than two weeks.)

This form lists 57 items related to the child’s sensory, behavioral, emotional, and language abnormalities. This form lists 57 items related to the child’s sensory, behavioral, emotional, and language abnormalities. Circle the number for those items most accurately describing the child. Do not miss any items.

Note: Sensory Ability (S), Relating Ability (R), Body & Object Use (B), Language Ability (L), and Social & Self-Help Ability (S)

**Item**	**Score**				
	S	R	B	L	S
Whirls self for long periods of time			4		
Learns a simple task but “forgets” quickly					2
Frequently does not attend to social/environmental cues		4			
Does not follow simple commands (sit down, come here, stand up) given once				1	
Does not use toys appropriately (spins wheels, etc.)			2		
Poor use of visual discrimination when learning (fixates on parts of objects such as size, colour, position)	2				
Lacks a social smile (may smile out of context)		2			
Exhibits pronoun reversal (For example, saying 'I' instead of 'you,' etc.)				3	
Insists on keeping certain objects with him/herself			3		
. Seems not to hear (despite normal hearing tests	3				
. Speech is atonal and arrhythmic				4	
12. Rocks self for long periods of time			4		
13. Does not (or did not as a baby) reach out when reached for		2			
14. Strong reactions to minor changes in routine / environment					3
. Does not respond to own name when called out among two or more other names				2	
. Lunges and darts about, interrupted by spinning, toe walking, hand flapping			4		
17. Not responsive to other people’s facial expressions or feeling		3			

18. Seldom uses “yes” or “I”				2	
Has special abilities in one area -seems to rule out mental retardation					4
Does not follow simple prepositional commands (e.g., “put the ball in the box”)				1	
21. Sometimes shows no “Startle response” to a loud noise	3				
22. Flaps hands (or other self-stimulating behaviour)			4		
23. Severe temper tantrums and/or frequent minor tantrums					3
24. Actively avoids eye contact		4			
25. Resists being touched or held		4			
Sometimes, painful stimuli (cuts, injections, bruises) evoke no reaction	3				
27. Is (or was as a baby) stiff and hard to hold		3			
**Item**	**Score**				
	S	R	B	L	S
28. Is flaccid(doesn’t cling) when held in arms		2			
29. Gets desired objects by gesturing				2	
30. Walks on toes			2		
31. Hurts others by biting, hitting, kicking....					2
32. Repeats phrases over and over again				3	
33. Does not imitate other children at play		3			
34. Often will not blink when a bright light is directed towards eyes	1				
35. Hurts self by biting hand, banging head...			2		
36. Does not wait for needs to be met (wants things immediately)					2
37. Cannot point to more than five named objects				1	
38. Has not developed any friendships		4			
39. Covers ears at many sounds	4				
40. Twirls, spins, and bangs objects a lot			4		
41. Difficulties with toilet training					1
42. Uses 5 or less words per day spontaneously to communicate wants or needs				2	
43. Often frightened or very anxious		3			
44. Squints, frowns, or covers eyes when in the presence of natural light	3				
45. Does not dress self without frequent help					1
46. Repeats sounds or word over and over again				3	
47. “Looks through” people		4			
48. Echoes questions or statements made by other people				4	
Frequently unaware of surroundings and may be oblivious to dangerous situations					2
50. Prefers to manipulate and be occupied with inanimate objects					4
51. Will feel, smell, or taste objects in the environment			3		
52. Frequently had no visual reaction to a “new” person	3				
Gets involved in complicated “rituals” such as lining things up (for example, the child must walk a specific route, or before eating, sleeping, or doing something, they must place certain objects in certain positions or perform certain actions. Otherwise, they refuse to sleep, eat, etc.)			4		

Is very destructive (toys and household items are quickly broken)			2		
A developmental delay was identified at or before 30 months of age					1
Uses at least 15 but less than 30 spontaneous phrases daily to communicate				3	
57. Stares into space for long periods of time	4				
**Total**:					
**Overall Total:**					
**Comments:**					

Structure of the Scale and Scoring Standards: The ABC (Autism Behavior Checklist) consists of 57 items, covering symptoms related to sensory, behavioral, emotional, language, and self-care aspects of individuals with autism. These can be summarized into 5 factors:

- Sensory (S) (9 items, total of 26 points)
- Relating (R) (12 items, total of 38 points)
- Body and Object Use (B) (12 items, total of 38 points)
- Language (L) (13 items, total of 31 points)
- Social and Self-Help (S) (11 items, total of 25 points)

Each item is assigned a different score based on its weight in the scale, ranging from 1 to 4 points. For any item, as long as the child exhibits that behavior, regardless of the severity, the corresponding score is awarded. The final result is determined based on the total score of all items.

Method of Evaluating Results:

- Total score < 53 points: Negative for screening
- Total score ≥ 53 and ≤ 67 points: Positive for screening
- Total score ≥ 68 points: Can assist in diagnosing autism.

The higher the total score on the scale, the more severe the behavioral symptoms of autism.

Appendix 3 – Data Form (CSHQ)

Childhood Sleep Habits Questionnaire (CSHQ)

Name: Sex: Age:

Name of Person filling form: Relationship:

Contact:

Date Of Birth: ____Year____Month ____ Date

Date & time of filling form: ____Year____Month ____Date

The following statements are about your child's sleep habits and possible difficulties with sleep. Think about the past week in your life when you answer the questions. If last week was unusual for a specific reason, choose the most recent typical week. Unless noted, check Usually if it occurs 5 or 6 times a week, Sometimes if it occurs 2 to 4 times a week, Rarely if it occurs once a week or less than once a week.

BEDTIME

Write in your child's usual bedtime: Weeknights _____:_____ am/pm

Weekends _____:_____ am/pm

	Usually<br><br>(5-7times /Week)	Sometimes<br><br>(2-4 times /Week)	Rarely<br><br>(0-1 times /Week)
**1.**Child goes to bed at the same time at night			
**2.**Child falls asleep within 20 minutes after going to bed.			
3.Child falls asleep alone in own bed			
4.Child falls asleep in parent's or sibling's bed.			
5.Child falls asleep with rocking or rhythmic movements.			
6.Child needs special object to fall asleep (doll, special blanket, stuffed animal, etc.)			
7.Child needs parent in the room to fall asleep.			
8. Child go to bed voluntary at bedtime			
9.Child resists going to bed at bedtime			
10. Child struggles at bedtime (cries, refuse to stay in bed etc.)			
11. Child is afraid of sleeping in the dark.			
12. Child is afraid of sleeping alone.			
13. Child is afraid to pass away in sleep.			

SLEEP BEHAVIOUR

Child’s usual amount of sleep each day： __hours __mins

(combining nighttime sleep and naps)

	Usually<br><br>(5-7times /Week)	Sometimes<br><br>(2-4 times /Week)	Rarely<br><br>(0-1 times /Week)
14.Child sleeps too little			
15. Child sleeps too much			
16. Child sleeps the right amount			
17. Child's sleep quality is good			
18. Child's sleep quality is consistent			
19.Child wets the bed at night			
20.Child talks during sleep			
21.Child is restless and moves a lot during sleep			
22.Child sleepwalks during the night			
23.Child sleep in the same room as the parents (or caregiver)			
24.Child sleep in the same bed as the parents (or caregiver)			
25.Child moves to someone else’s bed during the night (Parents, brother, sister)			
26.Child has physical aches and pain during sleep			
If yes, location on body：			
27.Child grinds teeth during sleep			
28.Child snores badly			
29.Child seems to stop breathing during sleep			
30.Child snorts and/or gasps during sleep			
31.Child has trouble sleeping away from home (visiting relatives/vacation)			
32.Child complains of sleeping poorly			
33.Child awakens during the night screaming, sweating and inconsolable			
34.Child awakes alarmed by a frightening dream			

WAKING DURING THE NIGHT：

	Usually<br><br>(5-7times /Week)	Sometimes<br><br>(2-4 times /Week)	Rarely<br><br>(0-1 times /Week)
35.Child awakes once during the night			
36. Child awakes more than once during the night			
37. Child need help to fall back asleep after waking up at night			
38. Duration of time child wakes up at night is usually: hours mins			

MORNING WAKING：

Write in time of day child wakes up in the morning: weeknights _____:_____ am/pm

weekends _____:_____ am/pm

	Usually (5-7times /Week)	Sometimes (2-4 times /Week)	Rarely (0-1 times /Week)
39.Child wakes up by him/herself			
40.Child requires alarm clock to wake up			
41. Child wakes up in negative mood			
42.Adults or siblings wake child up			
43.Child has difficulty getting out of bed in the morning			
44.Child takes a long time to be alert in the morning			
45.Child wakes up very early			
46.Child has poor appetite in the morning			

DAYTIME SLEEPINESS:

47. Child show signs of napping during the day			
48.Child suddenly fall asleep during activities?			
49. Child appear fatigued			
50. In the past week, has the child been very sleepy or fallen asleep in the following situations?			
	Not sleepy	Very sleepy	Falls asleep
Watching TV			

Sleep Time

Weekday Sleep Time = Weekday morning wake-up time - night fall-asleep time

Weekend Sleep Time = Weekend morning wake-up time - night fall-asleep time

Average Sleep Time = (Weekday Sleep Time × 5) + (Weekend Sleep Time × 2) / 7

Score Entry:

When entering the score for each question,

1= Occasionally (0 times/week to 1 time/week.) OR Not sleepy

2 = Sometimes (2 times/week to 4 times/week) OR Very sleepy

3= Usually (5 times/week to 7 times/week) OR falls asleep

Score Conversion:

Higher scores indicate a higher risk of sleep problems. The conversion method for questions 1, 2, 3, 10, 11, and 26 is as follows: 1 = 3, 2 = 2, 3 = 1 (i.e., convert an original score of 1 to 3, keep a score of 2 unchanged, and convert a score of 3 to 1).

For questions 4, 5, 6, 7, 8, 9, 12, 13, 14, 15, 16, 17, 18, 19, 20, 21, 22, 23, 24, 25, 27, 28, 29, 30, 31, 321, and 322, the original input values remain unchanged."

Result Evaluation:

A total score higher than 41 on the Children's Sleep Habits Questionnaire (CSHQ) indicates poor sleep

quality.

Appendix 4 – Consent Form

PARTICIPANT INFORMATION SHEET AND CONSENT FORM

You are being invited to participate in a research study. Your participation in this study is entirely voluntary. Before you take part in this research study, the study must be explained to you and you must be given the chance to ask questions. Pease read the following content carefully, it will help you understand why the study is being conducted, the research procedures, and the potential benefits, risks, and discomforts that may arise from participation. If you agree to participate, please sign the consent form. You will be given a copy of this document.

If you are a parent or legal guardian giving consent for a child to participate in the study, please note that the word “you” refers to your child.

STUDY INFORMATION

Protocol Title:

Transcranial Pulsed Current Stimulation on Social Functioning and Sleep in Children with Autism Spectrum Disorder: A multi-center, double-blind, sham-controlled, randomized clinical trial

Approval Number: 202201 (v1.2)

Principal Investigator:

Dr Zhenhuan Liu

Senior Consultant, Head of Department

Division of Paediatric Neurorehabilitation,

Department of Paediatrics,

Nanhai Maternity and Children’s Hospital Affiliated to Guangzhou University of Chinese Medicine

1.Purpose of the Research Study

The purpose of this study is to find out if transcranial pulsed current stimulation (tPCS) improves social functioning and sleep in children with autism spectrum disorder (ASD).

2.Background of the Study

Over the past twenty years, research has shown that the prevalence of ASD is increasing. ASD core symptoms include problems with sociability, communication and repetitive behaviours. It is also often accompanied by conditions such as sleep disturbances ,childhood anxiety, hyperactivity, and emotional disorders,imposing a significant burden on both the patient and their families. The prevalence of ASD is between 0.7% and 1.2%. Severe

cases of autism can disrupt the lives of entire families. In our previous research, we found that using transcranial electrical stimulation can alleviate core symptoms and sleep problems in children with ASD, thereby improving the quality of life. Our research goal is to provide futher scientific evidence on the safety and efficacy of transcranial pulse current stimulation as a potential therapy for children with ASD.

This study will be conducted as a multi-center clinical trial in the following locations: Nanhai Maternal and Children's Hospital, Dongguan Maternal and Child Health Hospital, Zhanjiang Maternal and Child Health Hospital, Luoding Maternal and Child Health Hospital, Meixian District Hospital of Traditional Chinese Medicine, Guangzhou Angel Children's Hospital , Shenzhen Luogang Maternal and Child Health Hospital and Sichuang Special Needs Education School and Hospital. Approximately 340 voluntary participants are expected to participate. This study has been approved by the Foshan Science and Technology Bureau, Guangdong Provincial Health Commission, China. This study has been reviewed and determined to adhere to the principles of the Helsinki Declaration and conforms to medical ethics by the Research Ethics Committee of Nanhai Maternal and Children's Hospital affiliated to Guangzhou University of Chinese Medicine.

You were selected as a possible participant in this study because:

- You have been diagnosed to have ASD
- Aged between 3 and 14 years old
- You have sleep difficulty

You will be excluded if you meet any of the following conditions:

1.Under 3 years old or 14 years old and above.
2.Severe cognitive impairment, with a Wechsler Intelligence Scale (IQ) score of ≤35.
3.Comorbid epilepsy or history of epileptic disorders
4.Presence of severe psychiatric disorders such as schizophrenia and psychosis, or a family history of schizophrenia and psychosis.
5.Diagnosed with obstructive sleep apnea.
6.Skull defect or presence of severe scalp infection at the proposed site of stimulation.
7.History of craniotomy, presence of any ferromagnetic metal or implanted medical devices in the head or body, eg. cochlear implant, pacemaker or defibrillator, history of severe neurological disorders such as brain tumors and intracranial infection
8.History of substance abuse or dependence. Currently using benzodiazepines, neuroleptics, and antipsychotic medications such as Haloperidol 1mg/qd and Risperidone 5mg/qd. The introduction of new antipsychotic-related medications during the trial period is not allowed.
9. Currently participating in or having participated in other non-invasive brain stimulation treatments such as repetitive transcranial magnetic stimulation (rTMS) or transcranial direct current stimulation (tDCS) within the past 3 months.

3. Your Responsibilities in this Study

If you agree to participate in this study, you should be prepared to undergo inquiries about your medical history and examinations to determine if you can participate. At the start of the study, a random number provided by a computer which will determine whether you will receive tPCS treatment. There is a 50% chance that you will be assigned to either one of two different treatment groups: real-tPCS or sham (placebo)-tPCS. Neither you nor your doctor will know or be able to choose which treatment method you receive in advance. Regardless of group assignment, you will need to undergo tPCS once a day, 20-min per session , 5 times a week (Monday to Friday), over 4 weeks in hospital, followed by 1 hour of standard behavioural therapy that includes Applied Behaviour Analysis (ABA), structured learning, play-based therapy and speech therapy. As part of participating in the study, you need to cooperate with the research study team to complete 3 questionnaires on social functioning and sleep, before the start of treatment, and at the end of the 4 week treatment. You need to keep your study appointments, if it is necessary to miss an appointment, please contact the study staff to reschedule as soon as you know you will miss the appointment. You will inform the Principal Investigator as soon as possible about any side effects that you may have encountered.

4. Potential Benefits of Participating in this Study

The use of tPCS may help to improve social functioning and sleep in a select group of patients with ASD. Previous research on this topic conducted domestically and internationally, have shown that tDCS and tPCS has positive effects on pediatric ASD patients. These treatments can alleviate core symptoms such as social functioning problems and reduce poor sleeping patterns, thereby enhancing the quality of life for you and your family. Your participation may add to the medical knowledge about the use of tPCS, and may help future development of the optimal treatment parameters for tPCS in the treatment of other children with similar conditions. You will receive a comprehensive and adequate medical care during the study period.

5. Possible Risks, Discomforts or Inconveniences

The use of tPCS may cause discomfort and tingling sensation to some people. Some people may have allergic skin reaction to the electrodes. If you are unable to tolerate the tPCS treatment or the standard behavioural therapy, please contact the Principal Investigator as soon as possible. If there are other unexpected situations such as a change in your condition, regardless of whether it is related to tPCS or behavioural therapy, you should also promptly inform your doctor. He or she will make a judgment and provide medical treatment. The doctor will do their utmost to prevent and treat any harm that may arise from this study. If an adverse event occurs during the clinical study, a medical expert committee will determine whether it is related to the study intervention and will cover the cost of treatment for study-related injuries. During the study, you will need to cooperate with the doctor's examinations and inquiries, which may cause you some trouble or inconvenience. Additionally, there is a risk that the treatment may not prove effective and your condition may continue to progress. During the study period, if the doctor determines that the treatment provided is ineffective, the study will be terminated prematurely. Please note that behavioural therapy such as ABA, structured learning, play-based therapy and speech therapy are the current standard treatment for patients with ASD, whether or not you choose to participate in this study.

6. Costs and Payments if Participating in this Study

If you take part in this study, the use of tPCS over 4 weeks will be performed at free-of-charge to you. These costs will be borne by study grants. If you follow the directions of the Principal Investigator of this research study and you are injured due to the research procedure given under the plan for the research study, our institution will provide you with the appropriate medical treatment. Payment for management of the normally expected consequences of your treatment will not be provided by the hospital. You still have all your legal rights. Nothing said here about treatment or compensation in any way alters your right to recover damages where you can prove negligence.

7. Confidentiality of Personal Information

Your participation in this study will involve the collection of Personal Data. "Personal Data" means data about you which makes you identifiable (i) from such data or (ii) from that data and other information which an organisation has or likely to have access. Examples of personal data include name, identity card, nationality, passport information, date of birth, and telephone number.

Personal Data collected for this study will be kept confidential. Your study records and medical records, to the extent required by the applicable laws and regulations, will not be made publicly available. Only the study team will have access to the personal data being collected from you. In the event of any publication regarding this study, your identity will remain confidential. However, the monitor(s), the auditor(s), the Research Ethics Committee, and the regulatory authority(ies) will be granted direct access to your original medical records and study records to verify study procedures and data, without making any of your information public.

The data will be used for the purpose of this research study only, unless you give permission for your data to be made available for future use in other research studies. For this purpose, consent for future research will be sought from you.

By signing the Consent Form, you consent to (i) the collection, access to, use and storage of your Personal Data for this study, and (ii) the disclosure of such Personal Data to our authorised service providers and relevant third parties as mentioned above.

8. Participants' Rights to Obtain Information

You may ask any questions about this study at any time. You will be provided with a phone number so that you can contact your doctor and/or the study team to have your questions answered. If you have any complaints about participating in the study, please contact the research ethics committee office. In the event of any new information becoming available that may be relevant to your willingness to continue in this study, you (or your legal representative, if relevant) will be informed in a timely manner by the Principal Investigator or his/her representative and will be contacted for further consent if required.

9. Voluntary Participation or Withdrawal from the Study

Participation in this study is entirely voluntary. You may refuse to participate in this study or withdraw from it at any time during the study. This will not affect your relationship with your doctor or result in any loss of medical care or other benefits. You do not have to participate in this study to receive treatment for your condition. If you withdraw from the study for any reason, we would like you to complete a questionnaire to state the reason. If the doctor deems it necessary, you may also be asked to undergo laboratory tests and physical examinations, which are beneficial to protecting your health. However, the data that have been collected until the time of your withdrawal will be kept and analysed. The reason is to enable a complete and comprehensive evaluation of the study. Your doctor or the Principal Investigator may terminate your participation in this study at any time if there is a failure to follow the instructions of the Principal Investigator and/or study staff, the Principal Investigator decides that continuing your participation could be harmful, you need treatment not allowed in the study or the study is cancelled. If you do not participate in this study or withdraw from it midway, there are other alternative medications and treatment methods available.

10. Your Decision

It is fully your decision whether to participate in this study. You may discuss it with your family or friends before making a decision. Before deciding to participate, please ask your doctor any questions you may have until you fully understand the study. Thank you for reading the above material. If you decide to participate in this study, please inform your doctor or research assistant, and he/she will arrange all matters related to the study for you.

CONSENT FORM FOR RESEARCH STUDY

Protocol Title:

Transcranial Pulsed Current Stimulation on Social Functioning and Sleep in Children with Autism Spectrum Disorder: A multi-center, double-blind, sham-controlled, randomized clinical trial

Principal Investigator:

Dr Zhenhuan Liu

Senior Consultant, Head of Department

Division of Paediatric Neurorehabilitation,

Department of Paediatrics,

Nanhai Maternity and Children's Hospital Affiliated to Guangzhou University of Chinese Medicine

I agree to participate in the research study as described and on the terms set out in the Participant Information Sheet. The nature, risks and benefits of the study have been explained clearly to me and I fully understand them.

I understand the purpose and procedures of this study. I have been given the Participant Information Sheet and the opportunity to discuss and ask questions about this study and am satisfied with the information provided to me.

I understand that my participation is voluntary and that I am free to withdraw at any time, without giving any reasons and without my medical care being affected.

I agree □ or refuse □ to allow my medical information to be used in other research beyond this stud

____________________ ____________________ ____________________

Name of participant Signature/Thumbprint Date of signing

To be completed by parent / legal guardian / legal representative, where applicable

I hereby give consent for ______________________ (Name of Participant) to participate in the research study. The nature, risks and benefits of the study have been explained clearly to me and I fully understand them.

____________________ ____________________ ____________________

Name of participant's Signature/Thumbprint Date of signing

parent/ legal guardian/

legal representative

To be completed by witness, where applicable

I, the undersigned, certify that:

- I am 21 years of age or older.
- To the best of my knowledge, the participant or the participant's legal representative signing this informed consent form had the study fully explained to him/her in a language understood by him/ her and clearly understands the nature, risks and benefits of the participant's participation in the study.
- I have taken reasonable steps to ascertain the identity of the participant or the participant's legal representative giving the consent.
- I have taken reasonable steps to ascertain that the consent has been given voluntarily without any coercion or intimidation.

Witnessed by: ________________________________ ____________________

Name of witness Date of signing

____

Signature of witness

Investigator's Statement

I, the undersigned, certify to the best of my knowledge that the participant/ participant's legal representative signing this consent form had the study fully explained to him/her and clearly understands the nature, risks and benefits of the participant's participation in the study.

________________________ ____________________ ________________

Name of Investigator/ Signature Date

Person obtaining consent

CHILD/ PARTICIPANT ASSENT FORM

STUDY INFORMATION

Protocol Title:

Transcranial Pulsed Current Stimulation on Social Functioning and Sleep in Children with Autism Spectrum Disorder: A multi-center, double-blind, sham-controlled, randomized clinical trial

Approval Number: 202201 (v2)

Principal Investigator:

Dr Zhenhuan Liu

Senior Consultant, Head of Department

Division of Paediatric Neurorehabilitation,

Department of Paediatrics,

Nanhai Maternity and Children's Hospital Affiliated to Guangzhou University of Chinese Medicine

You are being asked to be in a research study. This is because you have Autism Spectrum Disorder (ASD), a brain condition that is related to problems with socializing, communication and behaviours. This study will look at a new experimental medical device, the AscenZ-IV Stimulator. We want to see how well it works and if it is safe, that is why you have been asked to participate.

If you say yes, you will have to do certain things, like:

- Complete questionnaires about your socializing, communication, behaviours and sleep quality, at two timepoints, i.e. at the beginning, and at Week four of the study.
- Come to the hospital to use the AscenZ-IV Stimulator for 20 minutes a day, 5 times per week, followed by an additional 1 hour per day of behavioural therapy that includes Applied Behaviour Analysis (ABA), structured learning, play-based therapy and speech therapy
- Record any adverse events during the 4 weeks of treatment.

The AscenZ-IV Stimulator applies the electrical currents to the scalp. The use of the device may help reduce sociability, communication and sleep issues, and make you feel better.

ASSENT

This research study has been explained to me and I agree to be in this study

______________________________ ______________________

Child/ Participant Name for Assent Date

Appendix 5 – Data & Safety Monitoring Committee

Composition of DSMC:

The purpose of the Data and Safety Monitoring Committee is to ensure the safety and well-being of participants, and the integrity of the data collected for the study. Formation of this committee includes the Principal Investigator, Dr Liu Zhen Huan and experts who are not part of the study team within the pediatric department and other departments from different participating site.

Name	Title	Department	Institution
Zhenhuan Liu	Senior Consultant , Head of Department	Paediatrics	Nanhai Maternity and Children's Hospital
Li Li Hu	Senior Consultant, Child Psychologist	Psychiatry	Meixian District Hospital of Traditional Chinese Medicine
Wen Na Fang	Senior Therapist	Pediatrics Neurorehabilitation	Nanhai Maternity and Children's Hospital
Bihui Pang	Senior Consultant	Pediatrics	Nanhai Maternity and Children's Hospital
Xiao Yang Lin	Senior Consultant, Chief Paediatric Acupuncturist	TCM Acupuncture	Dongguan Maternal and Child Health Hospital
Wen Jing Liu	Associate Consultant	TCM Acupuncture	Dongguan Maternal and Child Health Hospital
Yue Lan	Senior Consultant, Paediatric Neurologist	Paediatrics	Zhanjiang Maternal and Child Health Hospital

A5.1 Roles and responsibilities:

The Principal Investigator will ensure adherence with the protocol, regulatory standards and ethical guidelines, as well accuracy in relation to data entry. The P.I will also monitor safety adverse events reported during the study, according to safety monitoring plan specific in the trial protocol section 7. The P.I will perform data and safety monitoring through making regular contact with the relevant personnel in charge of on-site monitoring at each of the participating sites, through phone calls and WeChat messaging and email. In addition, the P.I will make no less than one trip every 4-6 months, in person, to each of the participating sites within the study period, to ensure that study data is authentic, complete and the data entry accurately correlates with the hardcopy data forms.

The Principal Investigator will provide an update of findings to DSMC via a monthly report, disseminated to all members of the DSMC via email and/or WeChat for decision. The monthly report will include data integrity reports and summary SAEs reports from each site that identify any emerging trends or safety concerns. Emergency meetings may be called if it is in relation to safety adverse events. Thorough records of all monitoring activities, findings, and actions taken will be kept.

A5.2 Interim analysis:

Interim analyses will be performed when data collection has been completed for 50% (170 out of the 340) targeted subjects, with 85 in each group.

A5.3 Statistical interim analyses:

Primary outcome measure: Autism Treatment Evaluation Checklist (ATEC) total and subdomain scores will be used for analysis of change in social functioning. T-tests, ANOVA and ANCOVA will be used to examine the rate of improvement for these scores. Scores will be compared with baseline. Estimates of the difference in the average rate of improvement in the scores will be obtained. These estimates will then be tested against the null value of no difference between both groups.

Secondary Outcome measures: Autism Behaviour Checklist (ABC) total and subdomain scores will be used for analysis of change in social functioning. The Childhood Sleep Habits Questionnaire (CSHQ) will be used for analysis of change in sleep functions. Scores will be compared with baseline. Estimates of the difference in the average rate of improvement in the scores will be obtained. These estimates will then be tested against the null value of no difference between both groups.

Participant characteristics at baseline (including demographic and clinical) will be compared within each group using independent sample t-tests or chi-square test as appropriate for the variable.

A5.4 Interim Analyses Reporting:

The P.I will be responsible to present interim findings to the DSMC, the trial sponsor and regulatory health authorities of Foshan Science and Technology Bureau, Guangdong Provincial Health Commission, China.

A5.6 Trial Audit:

Members of the DSMC committee will meet in person, or virtually over Tencent Web Conference , once in every 6 months, to discuss findings from the monthly reports and interim report submitted by the P.I. Decisions taken will be by majority vote of the committeee, independent from the sponsor and investigators.
