# Supplementary material for: Transcranial Pulsed Current Stimulation and Social Functioning in Children With Autism: A Randomized Clinical Trial
Source: JAMA Netw Open. 2025 Apr 21;8(4):e255776. doi: 10.1001/jamanetworkopen.2025.5776 (PMC12013354; doi:10.1001/jamanetworkopen.2025.5776)
Supplement: Supplement 2. — eTable 1. Summary List of Adverse Events Across the Right Participating Sites eTable 2. Summary of Participants Achieving Clinically Meaningful Change in Post-hoc ATEC Total Scores eTable 3. Summary of Participants Achieving Clinically Meaningful Change in Post-hoc ABC Total Scores eFigure 1. 3D Head Model from T1-Weighted MRI of a 6-Year-Old to Simulate tPCS-Induced Electric Fields eFigure 2. Scatterplot Showing CSHQ Total Score Change Predicting ATEC Total Score Change for All Participants Post-Treatment [file jamanetwopen-e255776-s002.pdf]

Supplemental Online Content

Liu Z, Zhong S, Ho RCM, et al. Transcranial pulsed current stimulation and social functioning in children with autism: a randomized clinical trial. *JAMA Netw Open*. 2025;8(4):e255776. doi:10.1001/jamanetworkopen.2025.5776

eTable 1. Summary List of Adverse Events Across the Right Participating Sites

eTable 2. Summary of Participants Achieving Clinically Meaningful Change in Post-hoc ATEC Total Scores

eTable 3. Summary of Participants Achieving Clinically Meaningful Change in Post-hoc ABC Total Scores

eFigure 1. 3D Head Model from T1-Weighted MRI of a 6-Year-Old to Simulate tPCS-Induced Electric Fields

eFigure 2. Scatterplot showing CSHQ Total Score change Predicting ATEC Total Score Change for All Participants Post-Treatment

This supplemental material has been provided by the authors to give readers additional information about their work.

© 2025 Liu Z et al. *JAMA Network Open*.

eTable 1: Summary List of Adverse Events across the eight participating sites

Mild Adverse Events	Week 1		Week 2		Week 3		Week 4	
	Active-tPCS (n=155)	Sham-tPCS (n=157)	Active-tPCS (n=155)	Sham-tPCS (n=157)	Active-tPCS (n=155)	Sham-tPCS (n=157)	Active-tPCS (n=155)	Sham-tPCS (n=157)
Skin redness/itchiness	6	0	4	0	8	0	9	1
Mild Headache	4	2	3	1	3	1	3	1
Trouble Sleeping	nil	nil	nil	nil	nil	nil	nil	nil
Scalp Pain	nil	nil	nil	nil	nil	nil	nil	nil
Neck Pain	nil	nil	nil	nil	nil	nil	nil	nil
Moderate Adverse Events	Week 1		Week 2		Week 3		Week 4	
	Active-tPCS	Sham-tPCS	Active-tPCS	Sham-tPCS	Active-tPCS	Sham-tPCS	Active-tPCS	Sham-tPCS
Nausea	nil	nil	nil	nil	nil	nil	nil	nil
Scalp burns	nil	nil	nil	nil	nil	nil	nil	nil
Severe Headache	nil	nil	nil	nil	nil	nil	nil	nil
Dizziness	nil	nil	nil	nil	nil	nil	nil	nil
Abnormal Urine test	nil	nil	nil	nil	nil	nil	nil	nil

Adverse Events (AE) were collated across all the 8 participating hospital sites. Subjects that experienced adverse events were only recorded as “1” if they had not been recorded in the previous weeks, to avoid duplication. An AE is classified as “mild” , if they self-resolved and/or did not require further medical action. An AE is classified as “moderate” if they require medical intervention. An AE is classified as “serious” if it results in death, is life-threatening, requires inpatient hospitalization or prolongation of existing hospitalization or results in persistent or significant disability/incapacity.

© 2025 Liu Z et al. *JAMA Network Open*.

eTable 2: Participants Achieving Clinically Meaningful Change in Post-hoc ATEC Total Scores

ATEC * Change in ATEC Crosstabulation						
			Change in ATEC			Total
			Total score reduction >=10%	Total score reduction 0 to <10%	Total score without reduction	
ATEC	Sham-tPCS Group	Count	48	65	44	157
		Expected Count	66.4	54.3	36.2	157.0
		% within ATEC	30.6%	41.4%	28.0%	100.0%
	Active-tPCS Group	Count	84	43	28	155
		Expected Count	65.6	53.7	35.8	155.0
		% within ATEC	54.2%	27.7%	18.1%	100.0%
Total		Count	132	108	72	312
		Expected Count	132.0	108.0	72.0	312.0
		% within ATEC	42.3%	34.6%	23.1%	100.0%

Chi-Square Tests			
	Value	df	Asymptotic Significance (2-sided)
Pearson Chi-Square	17.843[a]	2	<.001
Likelihood Ratio	18.029	2	<.001
Linear-by-Linear Association	14.213	1	<.001
N of Valid Cases	312		
a. 0 cells (0.0%) have expected count less than 5. The minimum expected count is 35.77.			

© 2025 Liu Z et al. *JAMA Network Open*.

eTable 3: Participants Achieving Clinically Meaningful Change in Post-hoc ABC Total Scores

ABC * Change in ABC Crosstabulation						
			Change in ABC			Total
			Total score reduction >=10%	Total score reduction 0 to <10%	Total score without reduction	
ABC	Sham-tPCS Group	Count	31	63	63	157
		Expected Count	47.3	64.4	45.3	157.0
		% within ABC	19.7%	40.1%	40.1%	100.0%
	Active-tPCS Group	Count	63	65	27	155
		Expected Count	46.7	63.6	44.7	155.0
		% within ABC	40.6%	41.9%	17.4%	100.0%
Total		Count	94	128	90	312
		Expected Count	94.0	128.0	90.0	312.0
		% within ABC	30.1%	41.0%	28.8%	100.0%

Chi-Square Tests			
	Value	df	Asymptotic Significance (2-sided)
Pearson Chi-Square	25.313[a]	2	<.001
Likelihood Ratio	25.944	2	<.001
Linear-by-Linear Association	25.077	1	<.001
N of Valid Cases	312		

a. 0 cells (0.0%) have expected count less than 5. The minimum expected count is 44.71.

© 2025 Liu Z et al. *JAMA Network Open*.

e-Figure 1. An individualized 3D head model created from T1-weighted MRI scans of a 6-year-old to simulate tPCS-induced electric fields.

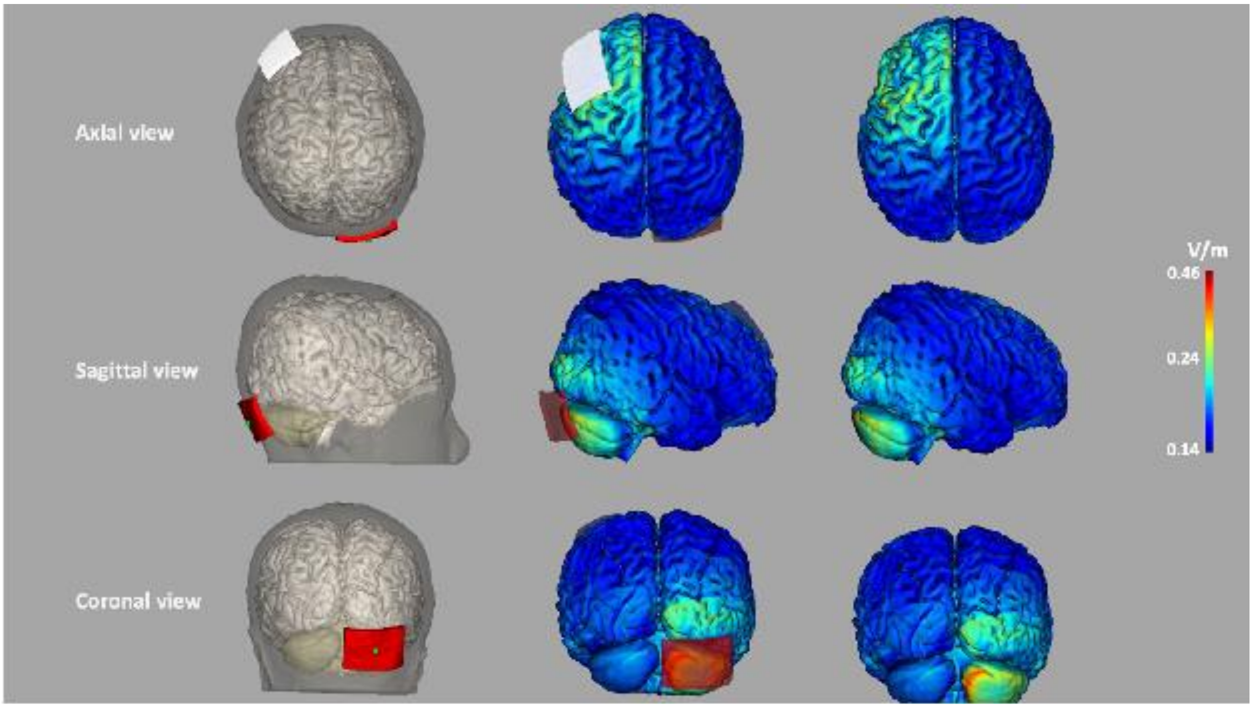

The MRI was segmented into various tissues, each with specific conductivities: gray matter (0.265 S/m), white matter (0.126 S/m), cerebrospinal fluid (1.65 S/m), ventricles (1.65 S/m), skull (0.01 S/m), and scalp (0.465 S/m). FEM simulations was used to estimate electric field distribution

© 2025 Liu Z et al. *JAMA Network Open*.

eFigure 2: Scatterplot showing CSHQ Total Score change predicting ATEC Total Score change for all participants post-treatment.

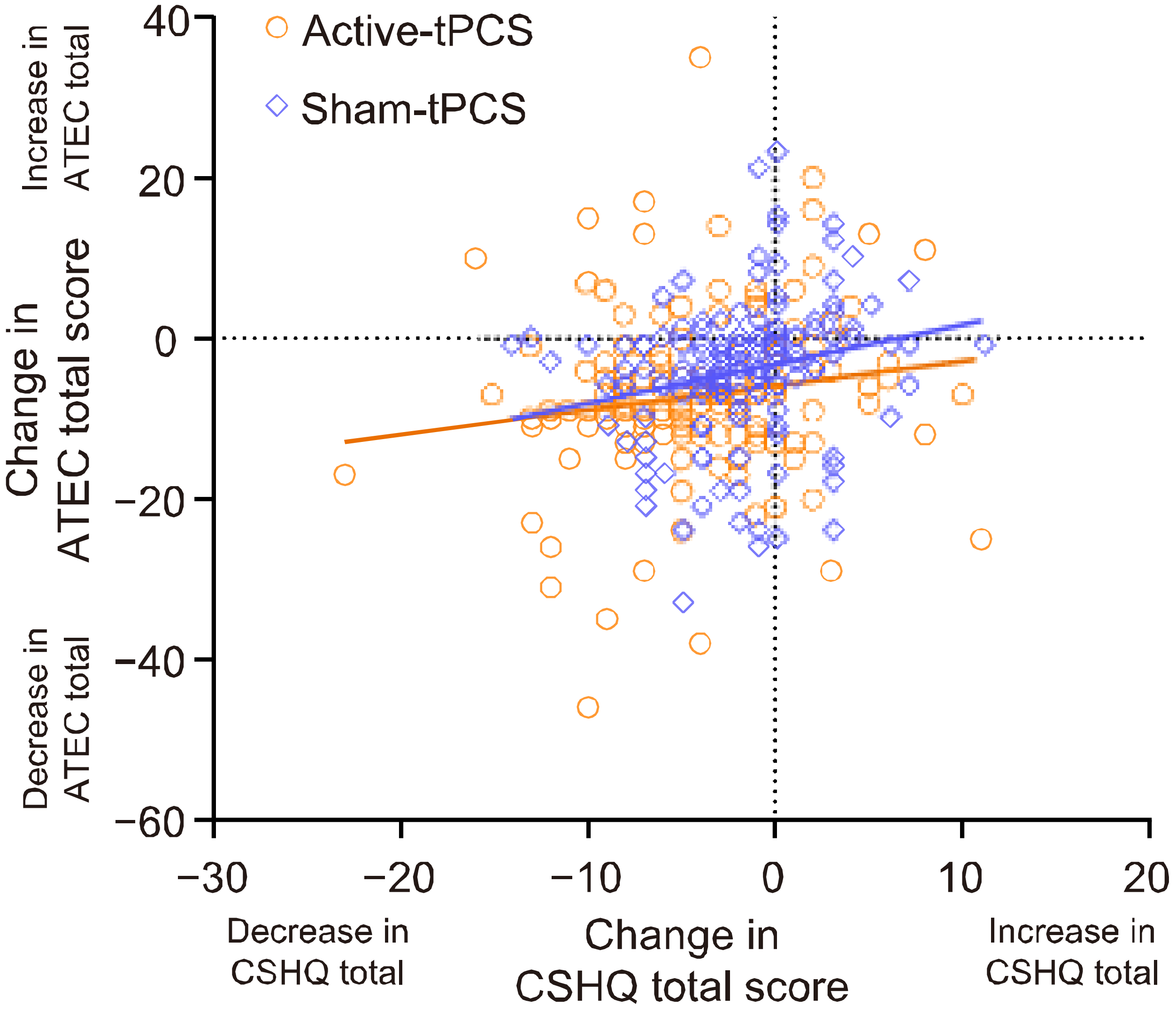

Exploratory post-hoc analysis revealed that the improvement in sleep (independent variable) did not significantly predict the improvement in social functioning (dependent variable) in the Active-tPCS group (Active-tPCS; R^2 linear = 0.023; *P* = 0.060).

© 2025 Liu Z et al. *JAMA Network Open*.
