# Supplementary material for: Transcranial Pulsed Current Stimulation and Social Functioning in Children With Autism: A Randomized Clinical Trial
Source: JAMA Netw Open. 2025 Apr 21;8(4):e255776. doi: 10.1001/jamanetworkopen.2025.5776 (PMC12013354; doi:10.1001/jamanetworkopen.2025.5776)
Supplement: Supplement 3. — Data Sharing Statement [file jamanetwopen-e255776-s003.pdf]

Data Sharing Statement

Liu. Transcranial Pulsed Current Stimulation and Social Functioning in Children With Autism. *JAMA Netw Open*. Published April 21, 2025. doi:10.1001/jamanetworkopen.2025.5776

Data
Additional Information: Chinese Clinical Trial Registry URL: https://www.chictr.org.cn/showproj.html?proj=166856 ChiCTR2200059118
Data available: Yes
Data types: Deidentified participant data
How to access data: The data has been uploaded on to ResMan Clinical Trial Management Public Platform http://www.medresman.org.cn/pub/cn/proj/projectshow.aspx?proj=11636
When available: With publication

Supporting Documents
Document types: Statistical/analytic code, Informed consent form
How to access documents: Request for documents may be sent to Dr. Liu Zhenhuan, email: lzh1958424@163.com
When available: With publication

Additional Information
Who can access the data: Researchers whose proposed use of the data has been approved
Types of analyses: For a specific purpose
Mechanisms of data availability: After approval of a proposal
